# Role of Abl Kinase and the Wave2 Signaling Complex in HIV-1 Entry at a Post-Hemifusion Step

**DOI:** 10.1371/journal.ppat.1000956

**Published:** 2010-06-17

**Authors:** Brooke Harmon, Nancy Campbell, Lee Ratner

**Affiliations:** Division of Molecular Oncology, Washington University School of Medicine, St Louis, Missouri, United States of America; University of Zurich, Switzerland

## Abstract

Entry of human immunodeficiency virus type 1 (HIV-1) commences with binding of the envelope glycoprotein (Env) to the receptor CD4, and one of two coreceptors, CXCR4 or CCR5. Env-mediated signaling through coreceptor results in Gαq-mediated Rac activation and actin cytoskeleton rearrangements necessary for fusion. Guanine nucleotide exchange factors (GEFs) activate Rac and regulate its downstream protein effectors. In this study we show that Env-induced Rac activation is mediated by the Rac GEF Tiam-1, which associates with the adaptor protein IRSp53 to link Rac to the Wave2 complex. Rac and the tyrosine kinase Abl then activate the Wave2 complex and promote Arp2/3-dependent actin polymerization. Env-mediated cell-cell fusion, virus-cell fusion and HIV-1 infection are dependent on Tiam-1, Abl, IRSp53, Wave2, and Arp3 as shown by attenuation of fusion and infection in cells expressing siRNA targeted to these signaling components. HIV-1 Env-dependent cell-cell fusion, virus-cell fusion and infection were also inhibited by Abl kinase inhibitors, imatinib, nilotinib, and dasatinib. Treatment of cells with Abl kinase inhibitors did not affect cell viability or surface expression of CD4 and CCR5. Similar results with inhibitors and siRNAs were obtained when Env-dependent cell-cell fusion, virus-cell fusion or infection was measured, and when cell lines or primary cells were the target. Using membrane curving agents and fluorescence microscopy, we showed that inhibition of Abl kinase activity arrests fusion at the hemifusion (lipid mixing) step, suggesting a role for Abl-mediated actin remodeling in pore formation and expansion. These results suggest a potential utility of Abl kinase inhibitors to treat HIV-1 infected patients.

## Author Summary

Patients infected with HIV-1 are currently treated with highly active antiretroviral therapy (HAART) that efficiently suppresses the virus but does not cure the infection. HIV-1 envelope activates Rac-mediated actin cytoskeleton rearrangements in the target cell that promote membrane fusion and entry. We discovered that these rearrangements require activation of the actin polymerization machinery including the tyrosine kinase Abl. We also showed that Abl kinase inhibitors imatinib, nilotinib, and dasatinib, current drug therapies for chronic myeloid leukemia, block HIV-1 entry and infection. These results suggest that these inhibitors might be appropriate drugs for treatment of HIV-1. This strategy of using inhibitors that disable host signaling proteins rather than viral proteins, essential for pathogen survival, may have a general efficacy in developing drugs to combat HIV-1 and other pathogens that acquire drug resistance.

## Introduction

HIV-1 enters cells in a pH-independent manner by fusion at the plasma membrane or from within endosomes [1]–[3]. HIV-1 entry requires multiple conformational changes in the HIV-1 glycoprotein, and rearrangement of the actin cytoskeleton. These events are triggered by binding of the viral envelope (Env) surface subunit gp120 to the primary receptor CD4 and one of two chemokine coreceptors, CCR5 or CXCR4 [1], [4]. This interaction activates signaling events in the cell, similar to those initiated by natural ligands, such as Ca^2+^ mobilization, activation of RhoGTPases, and phosphorylation of tyrosine kinases, pyk2, Zap70 and p56lck [4]–[6]. Rho family GTPases, which include the Cdc42, Rac, and Rho subfamilies, are responsible for regulating signaling from membrane receptors to the actin cytoskeleton. The Rho sub-family stimulates myosin based contractility, and drives the formation of stress fibers and focal adhesions. The Rac sub-family stimulates lamellipodia and membrane ruffles, and the Cdc42 subfamily stimulates the formation of filopodia [7]–[9]. We showed that HIV-1 Env binding to target cells induces activation of Rac, stimulates membrane ruffles and lamellipodia, and fusion is inhibited by dominant negative Rac [4], [10]. Furthermore, HIV-1 Env-induced Rac activation depends on activation of Gαq, phospholipase C (PLC), Ca^2+^ mobilization, protein kinase C (PKC), pyk2 and the GTPase Ras [5]. In the current study we identified the fusion-specific effectors of Rac required for actin cytoskeleton rearrangements that mediate membrane fusion and entry.

Guanine nucleotide exchange factors (GEFs) activate GTPases, facilitating the GDP to GTP switch, and regulate their downstream effects by participating in scaffolding protein complexes, thereby linking GTPase activity to specific effectors [7]–[9]. HIV-1 Env-induced Rac activation is mediated by a specific Rac GEF, either Tiam-1 or Trio [10], [11]. There are multiple effectors of Rac, including serine/threonine kinases, lipid kinases, actin-binding proteins, and adaptor/scaffold molecules [7], [12]. PAK is a downstream effector of Rac and Cdc42 that promotes stabilization of actin networks. Another downstream effector of Rac that nucleates actin polymerization is the Arp2/3 complex. The Arp2/3 complex is activated by the Wave2 complex through IRSp53, an adaptor protein that binds Rac and Wave2 [7]. The Wave2 complex includes Rac-associated protein 1, Nck-associated protein, Abl-interacting protein 2, and heat shock protein C300. Wave2 also associates with Abl, and Abl-mediated phosphorylation of Wave2 promotes its activation [13], [14]. In addition to determining which Rac effectors are critical for membrane fusion, we studied the steps in the membrane fusion process affected by these signaling molecules. These data demonstrate that the Wave2 signaling complex and Abl are required for Env-mediated membrane fusion, entry, and infection and that Abl kinase inhibitors arrest the fusion process at hemifusion.

## Results

### HIV-1 Env-Mediated Fusion Depends on the Wave2 Signaling Complex

**Figure 1:**
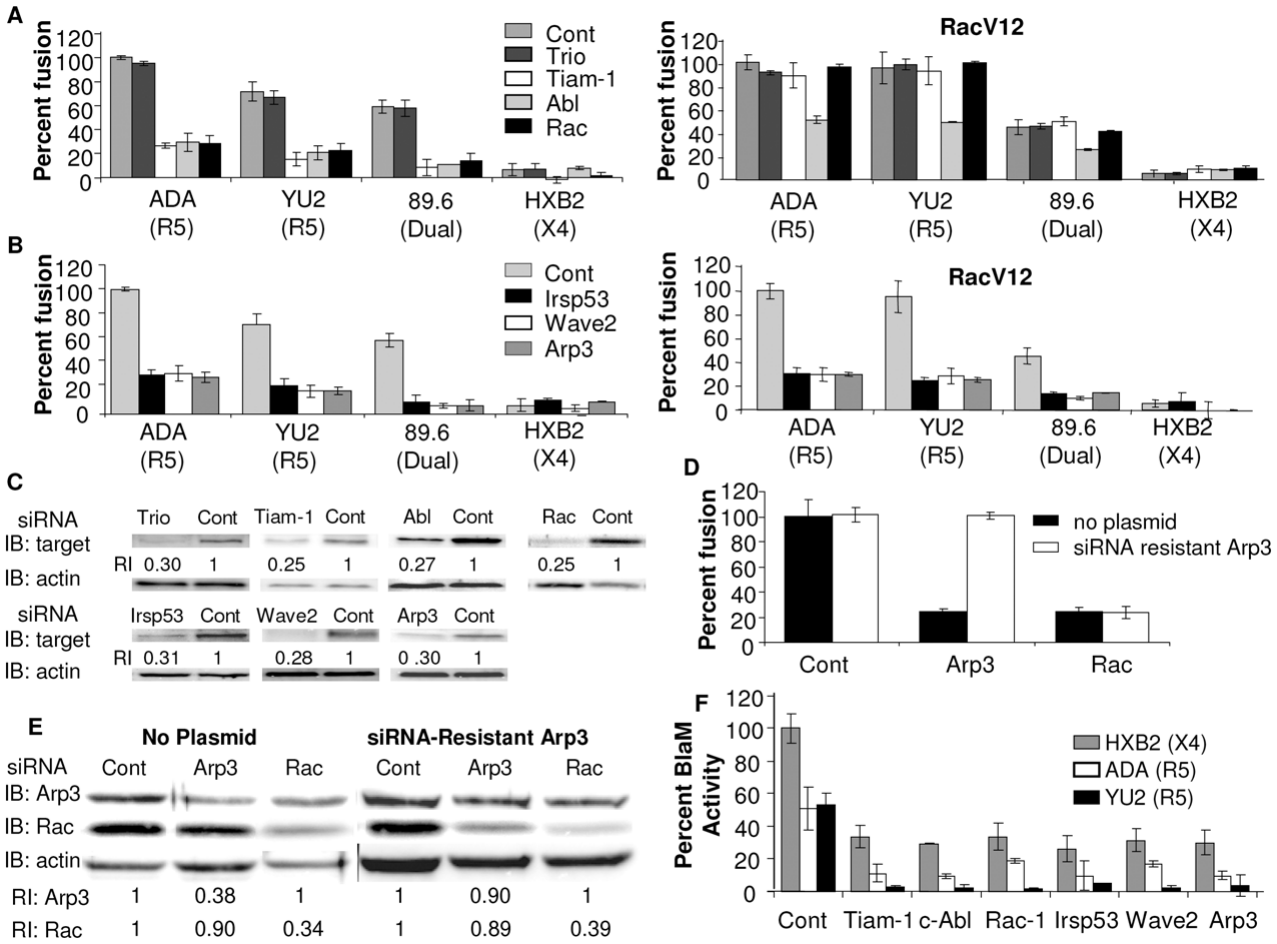
Down regulation of Wave2 signaling complex with siRNA reduces HIV-1 Env-mediated cell-cell fusion and virus-cell fusion. U87.CD4.CCR5 cells were transfected with control siRNA (control) or siRNA targeted against (A) Trio, Tiam-1, Abl, Rac, (B) IRSp53, Wave2, and Arp3. Cells were serum starved 24 h post-transfection (pt), infected with vCB21R alone or with vRacV12 48 h pt, and 72 h pt cells were incubated for 3 h with HIV_UNC_ (subtracted as background), HIV_ADA_, HIV_YU2_, HIV_89.6_ or HIV_HXB2_ Env-expressing cells and β-gal activity was measured (C) Each population of transfected cells was analyzed by Western blot with antibodies to the designated protein or actin. The relative reduction index (RI) is the quotient of the densitometry signal for the target band and that for actin, normalized by the ratio obtained with control siRNA. (D) U87.CD4.CCR5 cells engineered to express a siRNA resistant clone of Arp3 were transfected with control siRNA, or siRNA targeted against Arp3 or Rac. Cell fusion was measured by β-gal activity, and was normalized using control siRNA transfected cells incubated with HIV_ADA_ Env as 100%. (E) Western blots were performed on siRNA-resistant U87.CD4.CC5 cells expressing siRNA-resistant Arp3. (F) TZM-BL cells were transfected with 200 nM of targeted siRNA indicated and 48 h pt cells were incubated for 90 min with X4 HIV_HXB2_ virus R5 HIV_ADA_ virus or HIV_YU2_ virus. Fusion was stopped by adding lysis buffer with BlaM substrate. Cells were then incubated at rt overnight in the dark. OD values for no virus samples was subtracted as background and percent Blam activity was normalized using control siRNA transfected cells incubated with X4 HIV_HXB2_ virus as 100%. All data are representative of results from three similar experiments performed in triplicate.

To determine whether Abl, Trio, or Tiam-1 were required for HIV-1 Env-mediated cell-cell fusion, expression of these proteins was down regulated by RNA interference (RNAi) in U87.CD4.CCR5 cells. Cells expressing siRNA were then mixed with BSC40 cells expressing different Env subtypes and Env-dependent cell-cell fusion was measured. Transfection of target cells with siRNA to Tiam-1 and Abl decreased levels of Env-mediated cell-cell fusion by an average of 79±5% and 74±5% respectively for both HIV-1 R5 and dual-tropic Env-subtypes (Figure 1A, left). There was no significant fusion observed with CCR5 expressing target cells and X4 Env expressing cells with or without siRNA, as expected. The decrease in the levels of fusion correlated well with the decreased steady-state level of Tiam-1, and Abl as detected by immunoblot (Figure 1C). A siRNA directed against Trio had no effect on Env-induced cell-cell fusion despite a 70% reduction in expression of the Trio protein (Figure 1A and C). To determine whether Tiam-1 and Abl are acting exclusively upstream of Rac, a constitutively active Rac mutant, RacV12 was expressed in siRNA transfected cells. Expression of RacV12 in cells expressing siRNA to Tiam-1 reversed the effects of this siRNA on fusion, suggesting that Tiam-1 is functioning upstream of Rac. In contrast, levels of fusion in cells expressing RacV12 and siRNA to Abl were only 53±1% that of cells expressing RacV12 and control siRNA, suggesting a role for Abl upstream and downstream of Rac (Figure 1A, right).

Tiam-1 binds to the Rac and Cdc42 effector IRSp53, enhancing IRSp53 binding to Rac and activation of the Wave2 scaffolding complex [15]. To determine the role of these Rac effectors in Env-mediated membrane fusion, their expression was down regulated by RNAi in U87.CD4.CCR5 cells. The siRNA expressing cells were mixed with Env-expressing cells and cell-cell fusion was measured. Expression of siRNA to IRSp53, Wave2, and Arp3 decreased fusion by 74±5% 77±4% and 78±4%, respectively. The decrease in fusion with these siRNAs was not overcome by expression of RacV12, suggesting that these proteins are required downstream of Rac (Figure 1B). The decrease in levels of fusion correlated with the decrease in protein expression in cells expressing these siRNAs, as seen by immunoblot (Figure 1C), and each siRNA was specific for its target protein (Figure S1A). Treatment of cells stably expressing siRNA resistant Arp3, with Arp3 targeted siRNA had no effect on Env-mediated cell-cell fusion (Figure 1D, E). In contrast, with untransfected cells, and cells stably expressing siRNA resistant Arp3, treatment with siRNA to Rac decreased fusion by 75±5% and 76±3% respectively (Figure 1D). These results show that the effects of RNAi on fusion were specific to inhibition of their target molecules.

To demonstrate the role of Tiam-1, Abl, Rac, IRSp53, Wave2 and Arp3 in virus-cell fusion, their expression was down regulated by RNAi in TZM-BL cells, a derivative of HeLa cells that express CD4, CCR5, and CXCR4, and these cells were then used in a Vpr-Blam assay [16], [17]. In this assay siRNA expressing cells were mixed for 90 min with HIV-1 strains with cores carrying a β-lactamase (BlaM)-Vpr chimera, and pseudotyped with Env from ADA (R5), YU2 (R5) or HXB2 (X4), and fusion was quantified by measuring the cytosolic activity of viral core-associated BlaM [18]. Expression of siRNA to Tiam-1, Abl, Rac, IRSp53, Wave2, and Arp3 decreased virus-cell fusion by an average of 80±4%, 83±1%, 76±4%, 82±6%, 77±3% and 82±6%, respectively, for HIV-1 R5 and X4 Env subtypes (Figure 1F). These results show that activation of the Wave2 signaling complex is required for Env-dependent cell-cell fusion and virus-cell fusion.

### Small Molecule Inhibitors of Abl Kinase Activity Inhibit HIV-1 Entry

**Figure 2:**
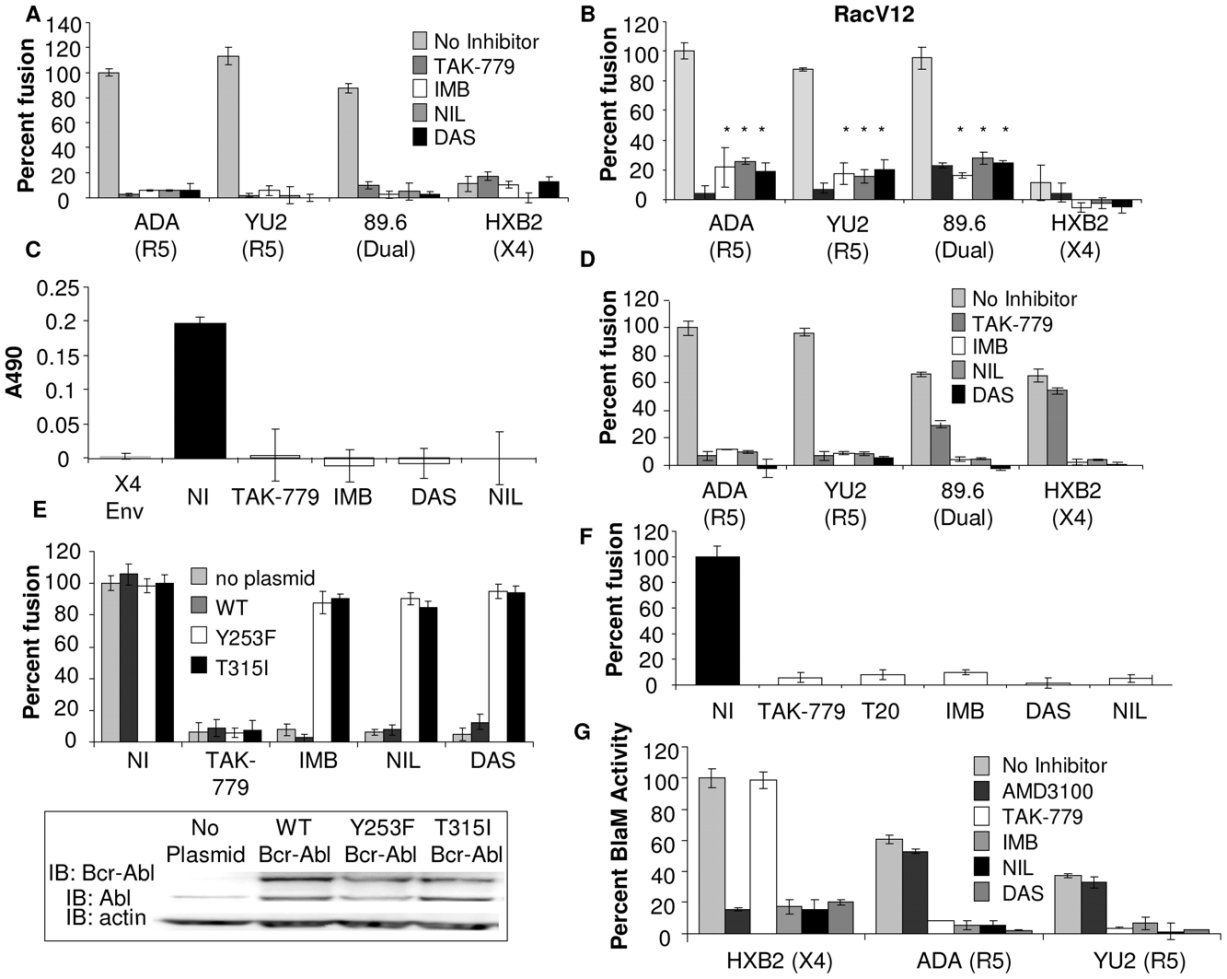
Abl kinase is required upstream and downstream of Rac for HIV-1 entry. (A) U87.CD4.CCR5 cells were infected with vCB21R alone, or with (B) vRacV12 overnight, then treated with DMSO alone, TAK-779, IMB, NIL, or DAS for 1 h and the inhibitors were also present during 3 h incubation with HIV-1 Env-expressing cells and β-gal activity as measured. (C) U87.CD4.CCR5 cells were treated for 1 h with TAK-779, IMB, NIL, or DAS and during 30 min incubation with BSC40 cells expressing no Env (subtracted as background), HIV_ADA_ Env or HIV_HXB2_ Env. Whole cell lysates were analyzed by Rac specific G-LISA activation assay. Average *A*490 of triplicate wells ± standard deviation are shown. (D) PBMCs were infected with vCB21R in complete media overnight, treated with DMSO, TAK-779, IMB, NIL, or DAS for 1 h prior to addition of HIV-1 Env-expressing cells and β-gal activity was measured. (E) U87.CD4.CCR5 cells engineered to express indicated clones of Bcr-Abl were treated with Abl inhibitors and HIV_UNC_ or HIV_ADA_ Env as described above or analyzed by Western blot with anti-Abl or anti-actin antibody (inset). (F) U87.CD4.CCR5 cells were infected overnight with vCB21R or vPT7-3, then mixed (1∶1) in triplicate wells, treated for 1 h with DMSO, TAK-779, T20, IMB, NIL, DAS, and with 100 ng of HIV_YU2_ for 3 h at 37°C. β-gal activity was measured and cell fusion was normalized using DMSO treated cells mixed with HIV_YU2_ as 100%. (G) TZM-BL cells were treated with DMSO, 1 µM TAK-779 or AMD3100, 10 µM IMB, 500 nM NIL, or 150 nM DAS for 1 h prior to 90 min incubation with indicated HIV viruses and BlaM activity was measured. Activity was normalized using DMSO treated cells mixed with HIV_HXB2_ virus as 100%. All data are representative of results from three similar experiments performed in triplicate.

Since treatment of cells with Abl targeted siRNA led to a decrease in Env-dependent cell-cell fusion and virus-cell fusion we wanted to determine whether treatment of target cells with commercially available Abl kinase inhibitors, imatinib (IMB), nilotinib (NIL), and dasatinib (DAS), block fusion. IMB is a relatively specific inhibitor of Bcr-Abl, Abl, Arg, and class III receptor tyrosine kinases. NIL is an Abl kinase inhibitor 20–50 fold more potent than IMB at inhibiting Abl. DAS, originally designed as a Src family kinase inhibitor, antagonizes Abl, ephrin and platelet-derived growth factor receptor kinases, and kit. DAS is 300 fold more potent than IMB at inhibiting Abl [19], [20]. To determine the concentrations of these Abl kinase inhibitors that inhibit Abl kinase activity and Env-mediated cell-cell fusion, without non-specific effects, Abl kinase activity, trypan blue analysis, vaccinia virus infection, and T7 polymerase activity were measured in addition to Env-dependent cell-cell fusion (Figure S1B, S2, and data not shown). Treatment of U87.CD4.CCR5 cells with 10 uM IMB, 500 nM NIL, and 300 nM DAS for 1 h prior to and during 3 h incubation with Env-expressing cells decreased Env-mediated cell-cell fusion by an average of 95±2%, 92±5%, and 92±6%, respectively, and Abl kinase activity by 85–87% (Figure 2A and S1B). The CCR5 inhibitor TAK-779, which completely blocks Env-mediated cell-cell fusion and infection of CCR5 expressing cells, was included as a control, and it decreased Env-dependent cell-cell fusion by 99±1% and Env-mediated Abl kinase activation by 98% (Figure 2A and S1B). Similar results were observed with U87.CD4.CXCR4 cells treated with CXCR4 inhibitor AMD3100 and Abl kinase inhibitors and incubated with cells expressing HIV-1 X4 or dual-tropic Env subtypes (Figure S3A). There was no decrease in T7 polymerase activity, or localization of CD4 and CCR5 on the cell surface (Figure S4 and data not shown). Expression of RacV12 in U87.CD4.CCR5 cells treated with IMB, NIL and DAS increased the level of fusion by an average of 3.5-fold (*, P<0.05) compared to treated cells without RacV12, suggesting a role of Abl kinase activity upstream of Rac (Figure 2B).

To determine the effect of these Abl kinase inhibitors on Env-induced Rac activation, U87.CD4.CCR5 cells were treated with inhibitors for 1 h prior to mixing with BSC40 cells expressing no HIV-1 Env, HIV-1 X4 Env, or HIV-1 R5 Env for 30 minutes in the presence of inhibitor. The mismatched X4 Env, that does not induce Rac activation in CCR5 expressing cells, and the CCR5 inhibitor TAK-779, which completely blocks Env-mediated Rac activation in CCR5 expressing cells, were included as controls [5]. Env-induced Rac activation was abolished in cells treated with TAK-779, and all three of the Abl kinase inhibitors (Figure 2C). To validate these effects in a relevant HIV-1 target cell, peripheral blood lymphocytes (PBLs), which express CD4, CCR5 and CXCR4, were used as the target cell population in an Env-dependent cell-cell fusion assay. Treatment of PBLs with IMB, NIL, and DAS decreased fusion by an average of 92±1%, 92±3%, and 99.5±1%, respectively, for HIV-1 R5, dual-tropic and X4 Env subtypes (Figure 2D). The CCR5 inhibitor TAK-779, as expected, completely blocked fusion mediated by R5 Env-expressing cells, inhibited fusion mediated by dual-tropic Env by 56±2%, and had no effect on fusion mediated by X4 Env (Figure 2D).

A long term infection assay was also performed where PBLs were infected with 150 ng of the X4 HIV_HXB2_ virus after 1 h preincubation with no inhibitor, DMSO, 10 µM IMB, 250 nM NIL, or 75 nM DAS. After 3 h, virus and inhibitors were washed off, inhibitors were added back and the plate was incubated at 37° for 21 days with addition of the inhibitors every 24 h. After 21 days the samples were assayed for cell viability and p24 antigen content. Treatment with IMB, NIL, and DAS decreased cell viability of HIV_HXB2_ infected cells by 17±4%, 8±5%, and 8±3% respectively and decreased infection by 52%, 51% and 94% compared to DMSO treated cells (Figure S5).

To validate the specificity of these effects, we performed an Env-dependent cell-cell fusion assay with cells stably expressing two different drug resistant Bcr-Abl mutants (Y253F and T315I), or expressing wild type (WT) Bcr-Abl [21]. Expression of the drug resistant Bcr-Abl mutants but not WT Bcr-Abl resulted in recovery of fusion (Figure 2E), demonstrating that the effects of these inhibitors on Env-dependent cell-cell fusion are specific to inhibition of Abl.

To confirm these results using virus particles with relevant levels of virus-associated glycoprotein, we used a virus-dependent cell-cell fusion assay based on the ability of virus particles to bridge two cells and allow transfer of cytoplasmic contents, and we also used the Vpr-BlaM assay described above [4], [10]. For the virus-dependent cell-cell fusion assay we used two populations of U87.CD4.CCR5 cells, one expressing the T7 polymerase and the other expressing the β-galactosidase (β-gal) gene under the T7 promoter. Both populations were incubated with inhibitors for 1 h prior to 3 h incubation with R5 virus HIV_YU2_. In this assay, controls included untreated and inhibitor treated cells that were not incubated with virus, the CCR5 inhibitor TAK-779, and T-20 which blocks entry by inhibiting the conformational change in HIV-1 gp41 required for fusion [17]. R5 Virus-dependent cell-cell fusion was reduced by an average of 94±3% in cells treated with IMB, DAS, and NIL compared to cells treated with DMSO alone, and treatment with TAK-779 and T-20 completely inhibited fusion (Figure 2F). Treatment of U87.CD4.CXCR4 cells incubated with the X4 virus HIV_HXB2_ with AMD3100 IMB, NIL, and DAS decreased virus-dependent cell-cell fusion by 88±7%, 98.6±1%, 87±5%, and 96±17%, respectively (Figure S3B).

For the Vpr-BlaM assay, TZM-BL cells were treated with 1 µM AMD3100, 1 µM TAK-779, 10 µM IMB, 500 nM NIL and 150 nM DAS for 1 hr prior to and during the 90 min incubation with HIV-1 Vpr-BlaM viruses expressing R5 and X4-tropic Env. AMD3100 treatment decreased X4-Vpr-BlaM activity by 84±1%, but had no effect on R5-Vpr-BlaM activity. TAK-779 treatment decreased R5-Vpr-BlaM activity by an average of 89±2%, but had no effect on X4-Vpr-BlaM activity, as expected. However, treatment of TZM-BL cells with IMB, NIL, and DAS decreased virus-cell fusion by an average of 81±4%, 89±5%, and 90±1%, respectively, for both HIV-1 R5 and X4 Env subtypes (Figure 2G). These results together with the results of the Env-dependent and virus-cell fusion assay demonstrate that Abl kinase is required for HIV-1 entry mediated by CXCR4 and CCR5.

### Infection of TZM-BL Cells with HIV-1 Particles, but not Particles Pseudotyped with Amphotropic Murine Leukemia Virus (A-MLV) Env or Vesicular Stomatitis Virus Glycoprotein (VSV-G), Depends on Abl and the Wave2 Signaling Complex

**Figure 3:**
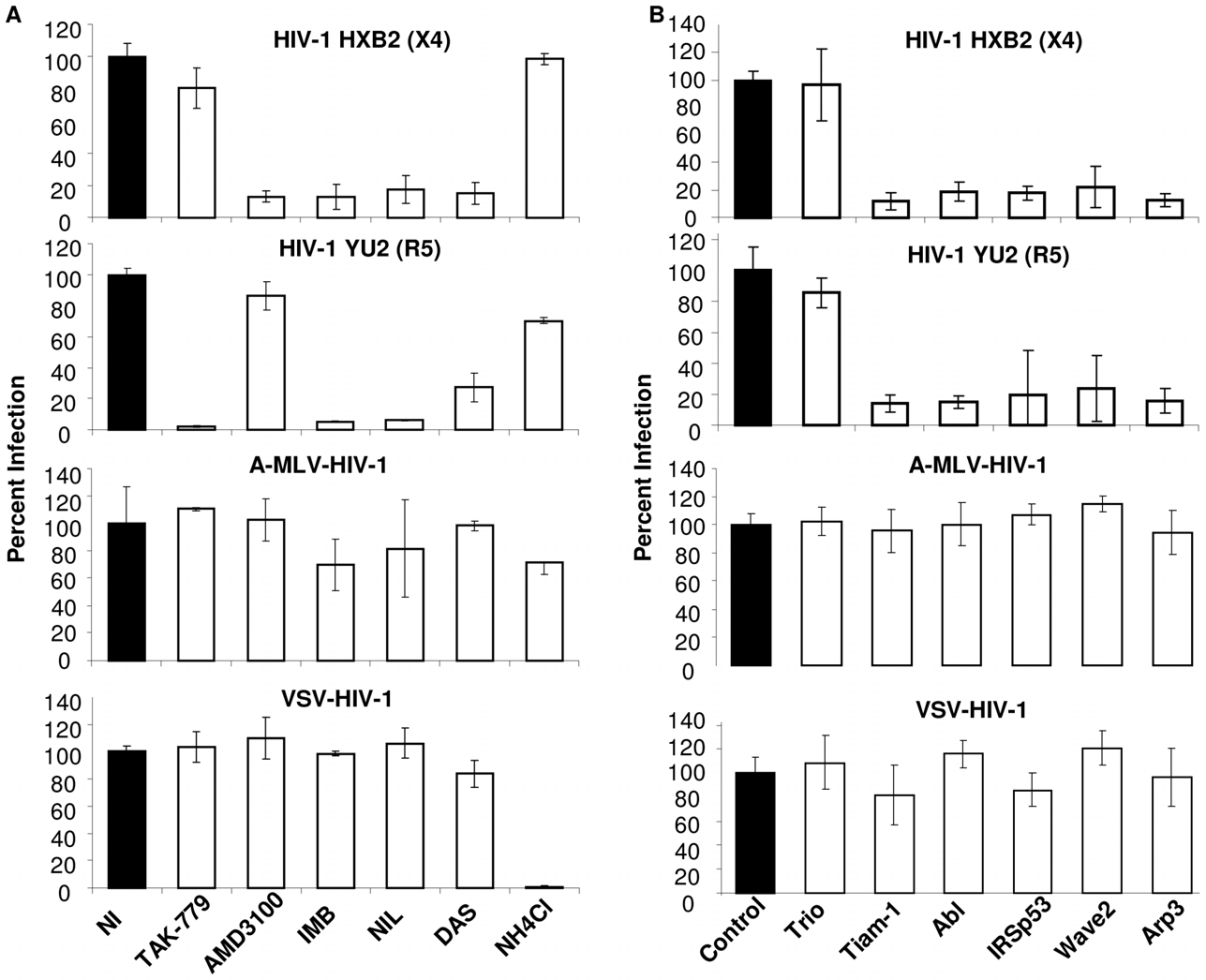
Infection with HIV-1 particles but not particles pseudotyped with MLV Env or VSV-G depends on the Abl and Wave2 signaling complex. (A) TZM-BL cells were incubated for 1 h with DMSO, TAK-779, AMD3100, NH_4_Cl, IMB, NIL, or DAS and 150 ng of HIV_HXB2_, HIV_YU2_, A-MLV-or VSV-G-HIV-1 per well was added for 3 h, washed, and cells were incubated with inhibitors overnight and luc activity was measured. (B) TZM-BL cells were transfected with control siRNA or siRNA directed against indicated target proteins and 48 h later infected with 150 ng of HIV_HXB2_, HIV_YU2_, A-MLV- or VSV-G-HIV-1 for 3 h, cells were washed and incubated overnight, and luc activity measured. Cell infection was normalized using (A) DMSO treated cells or (B) cells transfected with control siRNA as 100%. All data are representative of results from three similar experiments performed in triplicate.

To determine whether the Wave2 signaling complex and Abl are required exclusively for HIV-1 entry, or virus-induced fusion and infection in general, we examined infection with HIV-1 versus A-MLV Env (A-MLV-ENV-HIV-1) or VSV-G pseudotyped HIV-1 (VSV-G-HIV-1) using the TZM-BL assay. HIV-1 Env induces pH independent virus-cell fusion to facilitate entry, whereas viruses pseudotyped with VSV-G or A-MLV Env induce pH-dependent clathrin mediated endocytosis or caveolin-mediated endocytosis, respectively [22]–[25]. TZM-BL cells, a derivative of HeLa cells that express CD4, CCR5, CXCR4, and luciferase (luc) under the control of the HIV-1 LTR, were pretreated with the 10 µM IMB, 500 nM NIL and 150 nM DAS for 1 h prior to incubation with virus for 3 h, and a subsequent 24 h incubation with inhibitor only [16], [17]. The CCR5 inhibitor TAK-779, the CXCR4 inhibitor AMD3100, and ammonium chloride (NH_4_Cl) which inhibits endosomal acidification required for VSV-G mediated entry, were included as controls [22], [23], [26]. The top two panels of Figure 3A shows that treatment with IMB, NIL, and DAS decreased infection with R5 HIV_YU2_ virus and X4 HIV_HXB2_ virus by an average of 91±7%, 88±4%, and 91±5%, respectively, comparable to the reductions observed with Env-dependent cell-cell fusion, virus-dependent cell-cell fusion and virus-cell fusion (Figure 2). The Abl kinase inhibitors had no effect on infection of TZM-BL cells with A-MLV-ENV-HIV-1 or VSV-G-HIV-1, but treatment of cells with NH_4_Cl blocked infection with VSV-G-HIV-1 as expected (Figure 3A, bottom two panels). These data show that Abl-kinase inhibitors were able to block HIV-1 Env-mediated fusion specifically and had no effect on infection via pH-dependent clathrin-mediated or caveolin-mediated endocytosis, and post-entry steps were not affected by these inhibitors.

To test the effect of Wave2 complex targeted siRNAs on infection, TZM-BL cells were transfected with 200 nM control siRNA or siRNA directed towards Tiam-1, Trio, Abl, IRSp53, Wave2 and Arp3. These cells were incubated with virus for 3 h, and media alone for 24 h. The decreased levels of HIV-1_YU2_ and HIV-1_HXB2_ infection of TZM-BL cells expressing siRNA targeted to Tiam-1, Abl, IRSp53, Wave2, and Arp3 were comparable to levels of Env-mediated cell fusion with U87.CD4.CCR5 cells expressing these siRNAs, whereas siRNA to Trio had no effect (Figure 3B, top two panels). Steady state levels of target proteins in cells expressing targeted siRNAs were decreased to similar levels as in U87 cells (Figure 1C and data not shown). Infection of TZM-BL cells with A-MLV-ENV-HIV-1 or VSV-G-HIV-1 was not affected by expression of the targeted siRNAs, suggesting that Tiam-1, Abl, IRSp53, Wave2, and Arp3 are required for HIV-1 Env-mediated entry and are not necessary for post-fusion steps in the virus life cycle (Figure 3B, bottom 2 panels).

### The Abl Kinase Inhibitors Arrest Fusion at the Hemifusion Step

HIV-1 Env-induced fusion, and release of the viral capsid into the cytosol is a multistep process. First, gp120 binds to CD4 inducing conformational changes in gp120, and actin cytoskeletal rearrangements in the target membrane that bring the coreceptor CCR5 or CXCR4 into close proximity with CD4. Next, coreceptor binding to gp120 triggers conformational changes in gp41 to produce a prebundle conformation that inserts into the target cell membrane, allowing lipid mixing or hemifusion, and then pore formation. Additional conformational changes induce formation of the gp41 6-helix-bundle which prevents pore closure and facilitates pore enlargement and full fusion [2], [27], [28]. To determine which step(s) in the membrane fusion process are blocked by the Abl kinase inhibitors, we examined the effect on infection of membrane curving agents. Oleic acid (OLA), chlorpromazine (CPZ), and trifluoperazine (TFP) are lipid analogs that insert into the inner leaflet of the cell membrane. OLA induces negative curvature in the membrane that promotes formation of a hemifusion intermediate (i.e. lipid mixing), but cannot induce pore formation if there is a block at hemifusion. CPZ and TFP are membrane-permeable weak bases that partition into inner leaflets of cell membranes, induce positive curvature, and relieve a block at hemifusion [29]–[32].

**Figure 4:**
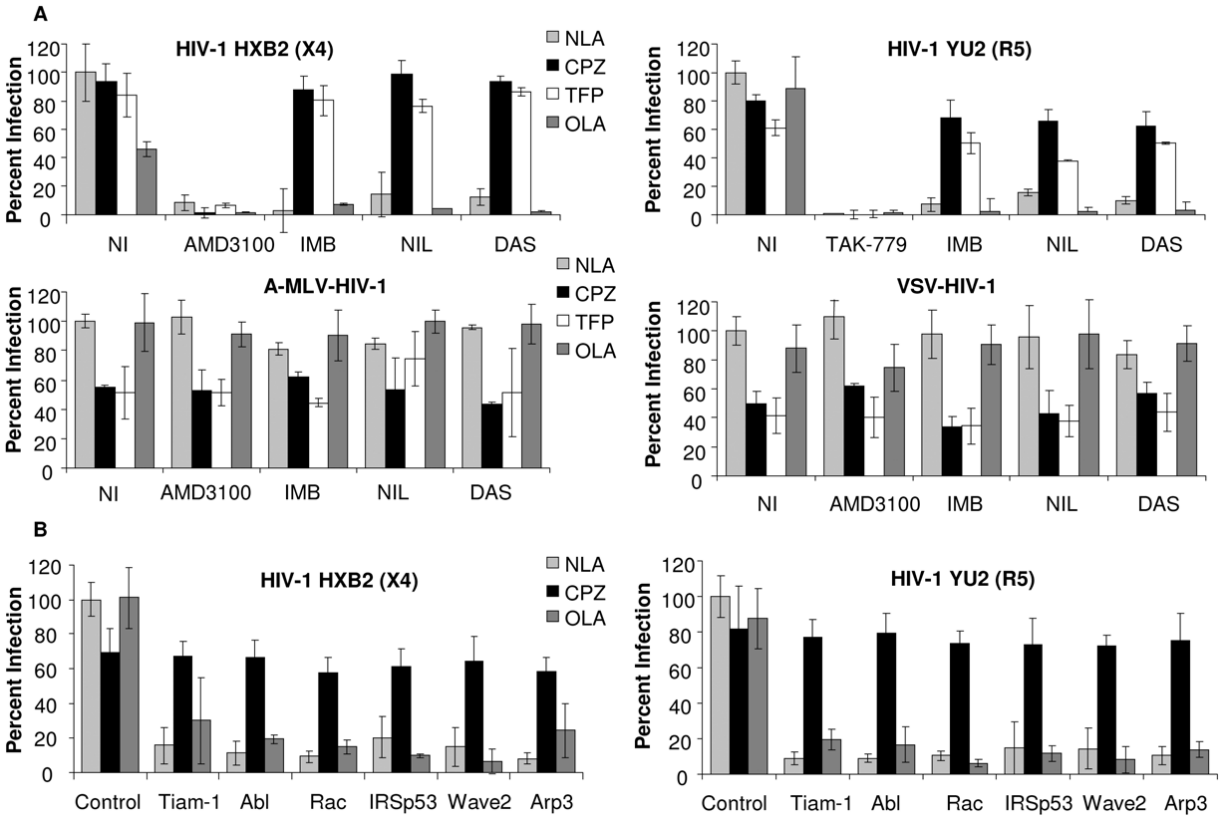
Abl kinase inhibitors and expression of siRNA targeted to the Wave2 complex block HIV-1 Env-mediated infection at a post-hemifusion step. (A) TZM-BL cells were treated with DMSO, TAK-779, AMD3100, IMB, NIL, or DAS for 1 h. HIV_YU2_ HIV_HXB2**,**_ A-MLV-Env-HIV-1 or VSV-G-HIV-1 (150 ng) was added for 1 h then cells were then treated with indicated lipid analogs for 1–5 min. Cells were washed and incubated in inhibitor overnight and luc activities were measured. (B) TZM-BL cells were transfected with 200 nM control siRNA or siRNA targeted to Tiam-1, Abl, Rac, IRSp53, Wave2 or Arp3 and 48 h pt cells were incubated with 150 ng of HIV_YU2_ or HIV_HXB2**.**_ After 3 h cells were washed and incubated at 37° in complete media overnight and luc activities were measured. Data are representative of results from three similar experiments performed in triplicate. Cell infection was normalized using DMSO treated cells or control siRNA transfected cells infected with HIV_YU2_ or HIV_HXB2_ as 100%.

To determine the effect of inhibitors and lipid analogs on HIV-1 infection, TZM-BL cells were treated with 1 µM AMD3100, 1 µM TAK-779, 10 µM IMB, 500 nM NIL, and 150 nM DAS for 1 h, prior to and during 1 h incubation with no virus, HIVΔ_ENV_, R5 HIV_YU2_, X4 HIV_HXB2_, A-MLV-ENV-HIV-1, or VSV-G-HIV-1. After 1 h, cells were treated with CPZ or TFP for 1 min or OLA for 5 min, followed by 2 h incubation with inhibitor and virus, and subsequent 24 h incubation with inhibitor only. Addition of CPZ and TFP to cells treated with Abl kinase inhibitors and infected with HIV_YU2_ or HIV_HXB2_ resulted in an 8 fold increase in infection compared to inhibitor treated cells infected in the absence of lipid analogs (Figure 4A), The exogenous cone shaped lipid OLA, which induces negative curvature of the membrane resulting in lipid mixing, had no affect on infection (Figure 4A). TAK-779 mediated inhibition of HIV_YU2_ infection and AMD3100 mediated inhibition of HIV_HXB2_ infection was not affected by these lipid analogs. No increase in luc activity was observed with lipid analog treatment of cells infected with HIVΔ_ENV_ versus no virus, indicating that Env is required to observe an increase in infection (Figure S6A). Treatment of A-MLV-ENV-HIV-1 and VSV-G-HIV-1 infected cells with CPZ and TFP decreased overall infection by 2 fold and had no effect on cells treated with Abl kinase inhibitors, indicating that the increase in HIV-1 infection observed with Abl kinase inhibitor treated cells was specific (Figure 4A, lower panels). CPZ also partially reversed the inhibitory effects of nilotinib as measured by the Vpr-BlaM assay (Figure S6B).

Similar increases in virus-dependent cell-cell fusion were observed when U87.CD4.CCR5 cells were treated with inhibitors and lipid analogs and HIV_YU2_ mediated fusion was measured after 3 h (Figure S6C). Cells were also incubated with the lipid analogs in the absence of HIV_YU2_ to account for the effects of these agents on the cells and on T7 polymerase activity. Addition of OLA did not increase fusion in cells treated with any of the inhibitors (Figure S6B). To confirm the results obtained with the Abl kinase inhibitors we incubated TZM-BL cells transfected with Tiam-1, Abl, Rac, IRSp53, Wave2, and Arp3 targeted siRNA, for 1 h with no virus, HIVΔ_ENV_, R5 HIV_YU2**,**_ or X4 HIV_HXB2_. After 1 h cells were treated with CPZ for 1 min or OLA for 5 min, followed by 2 h incubation with virus, and subsequent 24 h incubation with media alone. As with the Abl kinase inhibitors, treatment of siRNA transfected cells with CPZ increased infection by an average 8.4 fold compared to untreated cells, and OLA had no effect (Figure 4B). These results suggest that inhibition of Tiam-1, Abl, Rac, IRSp53, Wave2 or Arp3 arrests fusion at hemifusion, preventing pore formation, pore enlargement and content mixing.

**Figure 5:**
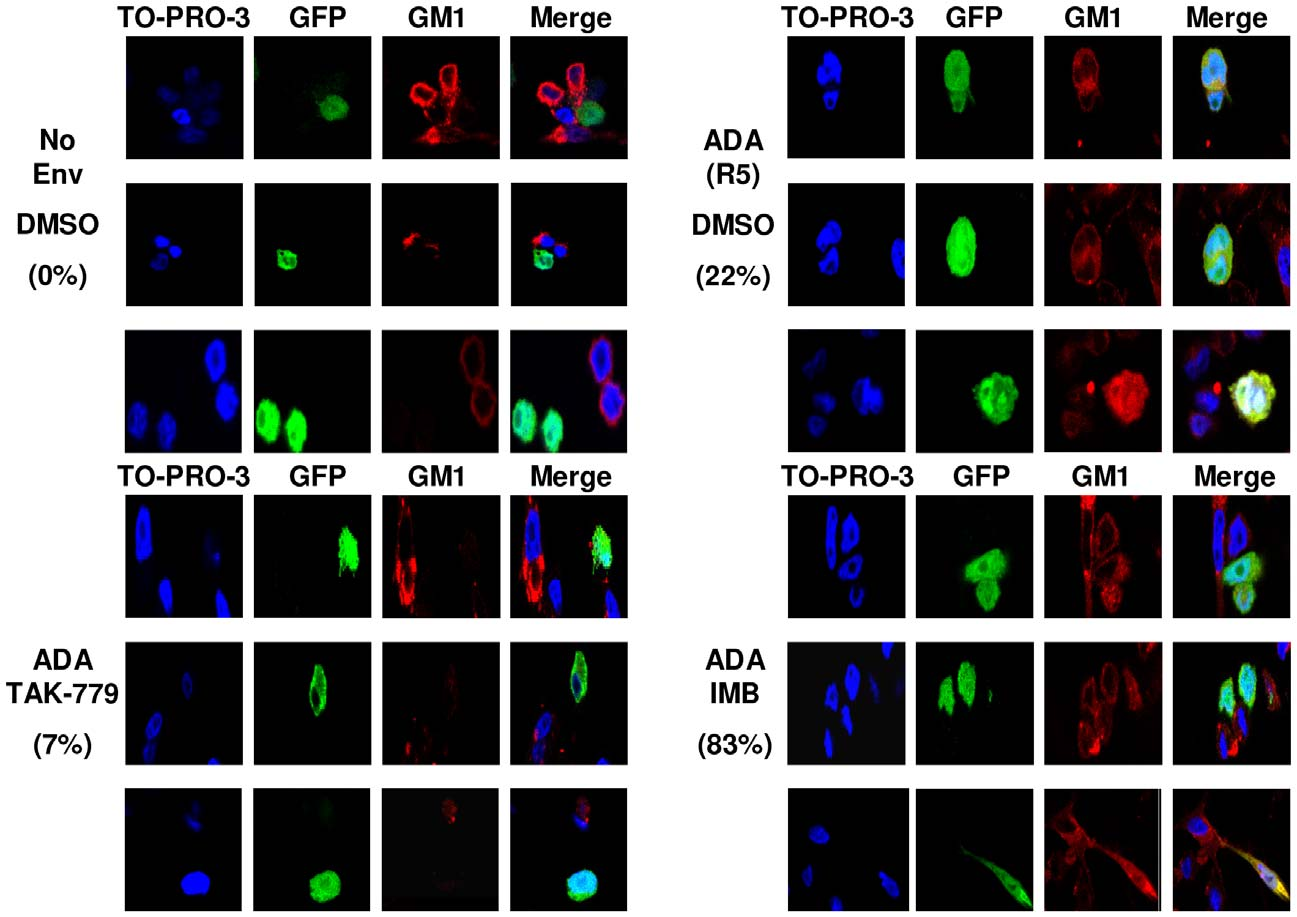
Abl kinase inhibitors arrest HIV-1 Env-mediated fusion at the hemifusion step. CHO-K1 cells that do not express GM1 were transfected with a GFP expressing plasmid (green), and 24 h later infected with WT vaccinia virus or vaccinia virus expressing HIV_ADA_ Env. After another 24 h, CHO-K1 cells were overlaid for 3 h with U87.CD4.CCR5 cells pre-treated for 1 h with DMSO, TAK-779, or IMB. Cells were fixed and stained with CTX-555 (red), and counterstained with TO-PRO 3 (blue). Images were collected using an oil objective (magnification X63). Images were cropped but relative cell size was maintained. The percentage of hemifused cells is listed.

To confirm that Abl kinase inhibitors cause arrest at hemifusion, we used a modification of a fusion assay described previously [33]. CHO-K1 cells that lack expression of the lipid ganglioside GM1, were engineered to express GFP and the HIV-1_ADA_ (R5) Env protein. U87.CD4.CCR5 cells were used as the target cell, and lipid mixing was detected when GM1, detected by a TRITC-conjugated form of cholera toxin β-subunit (CTX), was transferred from the target cell to CHO-K1-GFP cells. Complete fusion is detected when cells express GM1, GFP, and are multinucleated. Quantification was performed for three independent experiments and the percentage of hemifused GFP+, GM1+ cells with single nuclei and the percentage of multinucleated fully fused cells was enumerated for 68 cells from each condition (Figure 5, S7, and Table S1) There were 83.1±10.9% hemifused cells with IMB-treated cells mixed with HIV_ADA_-expressing CHO-K1 cells (Figure 5), compared to DMSO treated cells with 22.3±4.9% hemifused cells and 75.5±6.2% fully fused cells. With no HIV-1 Env or with the addition of TAK-779 there was little or no hemifusion or full fusion (Figure 5).

To demonstrate the effects of the lipid analog CPZ on HIV-1 Env mediated cell-cell fusion and to observe the effect of CPZ and the Abl kinase inhibitors on A-MLV Env or VSV-G induced cell-cell fusion we treated U87.CD4.CCR5 cells with DMSO, TAK-779, or IMB for 1 hr prior to incubation with CHO-K1 cells expressing no Env, HIV_ADA_, A-MLV Env or VSV-G for 1 hr. After 1 h cells were treated with CPZ for 1 min and OLA for 5 min, then washed and incubated with inhibitor for an additional 2 h prior to fixation and GM1 staining. Incubation of IMB treated cells with HIV_ADA_ and CPZ promoted the transition from hemifusion to full fusion as expected (Figure S8). Fusion of A-MLV Env and VSV-G Env expressing cells with U87.CD4.CCR5 cells was unaffected by treatment with IMB or CPZ (Figure S9) and all Env-mediated fusion was unaffected by OLA treatment (data not shown). These results confirm that Abl kinase activity is required at a post-hemifusion step for HIV-1 Env mediated fusion and entry.

## Discussion

Dynamic regulation of the actin cytoskeleton is required for fusion of biological membranes. Multiple reports have demonstrated that actin remodeling is required for HIV-1 mediated fusion and entry [4], [5], [10], [11], [34]–[36]. Some studies showed that treatment of target cells expressing physiologically relevant levels of receptor and coreceptor with the actin filament capping drug cytochalasin D prevented the formation of the gp120-CD4-coreceptor complex [35], [37], [38]. Another more recent study, demonstrated a role for CD4 and coreceptor-mediated filamin-A interactions in receptor clustering that is dependent on RhoA and ROCK mediated phosphorylation of ADF/cofilin [34]. Previous work from our lab with the actin filament stabilizing drug jasplakinolide and the actin monomer sequestering drug latrunculin A (LA) suggested a role for actin remodeling at a post binding step in fusion [4]. To further substantiate the role of actin polymerization in HIV-1 entry, we treated cells with 1 µM LA and 5 µM latrunculin B (LB). Both drugs blocked HIV-1 fusion for multiple cell types, as measured by the Env-dependent cell-cell fusion assay, the virus-dependent cell-cell fusion assay, the virus-cell fusion assay, and infection (Figure S10).

Our previous data demonstrated that the GTPase Rac was activated by HIV-1 Env ligation of CCR5, resulting in membrane ruffles and lamellipodia in the target cell membrane. Inhibition of this activation by dominant negative Rac or by a Rac GEF inhibitor completely abolished Env-dependent cell-cell fusion, virus dependent cell-cell fusion and infection [4], [5], [10], [11]. Our lab went on to show that Env-induced Rac activation is mediated by Gαq and its downstream effectors, including Ras. Other studies showed that Ras promotes Rac activation via direct interaction with Tiam-1, or by phosphatidylinositol 3-kinase (PI3K)-mediated activation of Tiam-1 [39]. Env-dependent Rac activation likely occurs through the first mechanism, since treatment of target cells with PI3K inhibitors had no effect on Env-dependent cell-cell fusion [40].

The nonreceptor tyrosine kinase, Abl, modulates actin upstream and downstream of Rac [41], [42]. In the current study, we used siRNAs and specific inhibitors to show that the activity of Abl kinase is required both upstream and downstream of Rac for Env-induced membrane fusion. Upstream of Rac, Abl phosphorylation of the Ras GEF complex promotes the activity of the Rac GEF Tiam-1, which was shown in the current study to be required for HIV-1 fusion. Downstream of Rac, Abl promotes phosphorylation and activation of Wave2 and its interaction with the Arp2/3 complex, events also demonstrated here to be critical for HIV-1 infection, but not VSV-G or A-MLV Env-mediated infection. These results suggest that these signaling mediators are important for HIV-1 Env mediated entry, are not necessary for pH dependent clathrin or caveolin-mediated endocytosis, and are not required at post-entry steps in the virus life cycle.

There is some conflict in the literature as to the location and mechanism of virus cell fusion. A recent report used microscopic imaging to track HIV-1 Env-pseudotyped MLV virus particles and observed virus-membrane fusion in endosomes [3]. This study also showed that virus-cell fusion and infection were inhibited in the presence of the dynamin inhibitor dynasore (DYN) which is known to block both clathrin and caveolin-mediated endocytosis [3]. The results in our current study suggest that fusion is occurring via a mechanism that is distinct from that of VSV (clathrin-mediated endocytosis) or A-MLV (caveolin-mediated endocytosis). In order to address this conundrum, we treated cells with the dynamin inhibitor DYN, and then used these cells for the Env-dependent cell-cell fusion assay, the virus-dependent cell-cell fusion assay, the virus-cell fusion assay and the TZM-BL infection assay. DYN treatment decreased HIV-1 Env-mediated infection and virus-cell fusion by an average of 58±7% and 50±3% respectively (Figure S10 and Figure S11). However treatment with DYN decreased A-MLV-Env-HIV-1 infection and VSV-G-HIV-1 infection by 75±5% and 89±1% respectively, showing that the affect on HIV-1 Env-mediated infection was not as significant (Figure S11). DYN treatment also decreased Env-dependent cell-cell fusion and virus-dependent cell-cell fusion by 53±8% and 50±10%, respectively which was unexpected since these assays both measure cell-cell plasma membrane fusion. Dynamins are a group of large GTPases that are involved in multiple processes in addition to endocytosis, such as vesicle transport, cytokinesis, organelle division, cell movement and cell signaling [43]–[45]. Therefore, the inhibition observed with the dynamin inhibitor DYN could be due to nonspecific effects on cellular processes. In support of this conclusion, a recent study used the Rev-dependent indicator cell line Rev-CEM to study the effects of DYN on HIV-1 replication and VSV-G-HIV-1 infection [44]. Using this assay they observed a dosage dependent decrease in VSV-G-HIV-1 infection with DYN treatment but did not see any decrease in HIV-1 infection [44]. These results as well as the results in Figure 3 show a clear distinction between HIV-1 Env-mediated entry and VSV-G- and A-MLV-mediated entry.

The current study also showed that the block in fusion caused by inhibition of Tiam-1, Abl, Rac, IRSp53, Wave2 and Arp3 occurs after hemifusion and before cytoplasmic mixing. This conclusion was based on the 1) confocal microscopy demonstration that addition of IMB to the fusion reaction allowed membrane but not cytoplasmic mixing, and 2) observation that lipid analogs that overcome a block at hemifusion overcame inhibition of HIV-1 virus dependent cell fusion, virus-cell fusion and infection caused by Abl kinase inhibitors and siRNA expression. These results support a model whereby HIV-1 Env binding to CCR5 stimulates activation of Gαq resulting in activation of Rac and activated Rac interacts with IRSp53. IRSp53 promotes Rac activation of the Wave2 complex, which is also activated by Abl, and activated Wave2 induces subsequent activation of Arp2/3-mediated actin rearrangements which facilitate pore formation, pore enlargement, and entry of HIV-1.

Many microbial pathogens depend on Abl family kinases to mediate efficient infection of their targeted host, including *Shigella flexneri*, enteropathogenic *Escherichia coli*, *Helicobacter pylori*, *Anaplasma phagocytophilum*, coxsackievirus, poxvirus, and murine AIDS virus. Abl kinases are involved in pathogen entry, intracellular movement, and exit from target cells; proliferation of target cells; and phosphorylation of microbial effectors. Many of these processes involve reorganization of the target cell actin cytoskeleton and depend on the same signaling pathways as HIV-1 [4], [5], [46]. Discovery of these signaling mediators as fundamental components of microbial pathogenesis provides new targets for therapeutic intervention. The clinical application of IMB, NIL, and DAS, which block deregulated Abl kinases in leukemia patients, demonstrate that inhibition *in vivo* is possible with manageable side effects [19], [20]. In addition IMB has been shown to be an effective inhibitor of anti-apoptotic pathways induced by HIV-1 in macrophages [47]. Most current antiviral therapies target viral proteins and mutation of the virus leads to therapy resistance. Therefore, using inhibitors that target host signaling proteins essential for HIV-1 entry may be an efficient new strategy for treatment of infected patients.

## Materials and Methods

### Reagents and Cell Lines

U87.CD4.CCR5 cells are astroglioma cells expressing CD4, CCR5-GFP or HA-CCR5. U87.CD4.CXCR4 cells are astroglioma cells expressing CD4 and CXCR4-GFP. CHO-K1 cells (ATCC) were grown in F-12K media with 10% serum and other cells maintained as described [48]. pMSCVneo-WT, Y253F, and T315I Bcr-Abl were gifts from Dr. R. Van Etten [21]. The siRNA resistant mutations were generated in Arp3 based on sequences obtained from Santa Cruz Biotechnology, Inc (SCBT, Santa Cruz, CA,), by PCR-mediated mutagenesis of a sub fragment that was sequenced to confirm the presence of mutations before sub cloning into the corresponding cDNA. WT and mutant cDNAs were cloned in pcDNA3.1^+^zeo for expression by transduction. IMB, NIL, and DAS were from LC Laboratories and were used at 10 uM, 500 nM, and 300 nM respectively unless indicated; CPZ (0.5 mM), TFP (0.3 mM), OA (100 nM), OLA (50 uM) and NH_4_Cl (50 mM) were from Sigma; TAK-779 (1 uM), and T-20 (10 ug/ml) were from the AIDS Research and Reference Reagent Program. The control siRNA constructs (non-targeting 20–25 nt siRNA designed as a negative control), the siRNA constructs and antibodies used for Western blots were from SCBT [5]. The siRNA constructs were transfected using GeneEraser siRNA Transfection Reagent or Lipofectamine RNAiMAX Transfection Reagent according to the manufacturer's instructions (Stratagene, La Jolla, CA, Invitrogen, Carlsbad, CA).

### Viruses

Wild-type (WT) vaccinia (WR strain) and recombinant vaccinia viruses expressing β-galactosidase (vCB21R), T7 polymerase (vPT7-3), constitutively active Rac GTPase (vRacV12), or HIV-1 Env proteins were described [48]. HIV with R5 YU2 or X4 HXB2 Env in HIV_NL4-3_ backbone were generated from 293T cells; some were pseudotyped with amphotropic murine leukemia virus (MLV) or vesicular stomatitis virus (VSV) glycoproteins [5]. TZM-BL assays were performed as described [5]. For the BlaM assay pseudoviruses were produced by co-transfecting 293T cells with HIV_NL4-3_ΔVpr expressing YU2, ADA, or HXB2 Env and BlaM-Vpr expressing pMM310 vector. Transfected 293T cell supernatants were harvested 48 h postlipofection, filtered, and assayed for p24 antigen content by enzyme-linked immunosorbent assay. Viruses were resuspended in culture media, aliquoted and stored at −80°C.

### BlaM Assay for Virus-Cell Fusion

TZM-bl cells were serum starved for 24–36 h then plated (4×10^4^ cells/well) in 96-well plates in complete media overnight. Cells were treated with indicated concentrations of inhibitors for 1 hr prior to and during 90 min incubation with DEAE-dextran (20 µg/ml) alone or DEAE-dextran (20 µg/ml) and 150 ng p24 HIV_YU2_Vpr-BlaM, HIV_ADA_Vpr-BlaM, or HIV_HXB2_Vpr-BlaM. After 90 min virus and media were aspirated off cells and 100 ul 1X Lysis and Detection Solution was added to wells (LyticBlazer-BODIPY FL, Invitrogen). The plate was incubated at room temperature in the dark overnight. The BlaM activity was quantified using TECAN fluorescence plate reader (Tecan, Switzerland). The extent of virus-cell fusion was measured with excitation centered at 485 nm and emission centered at 535 nm. The green signal for samples incubated with no inhibitors or inhibitors and no virus was subtracted as background from their respective virus treated samples.

### TZM-BL Assay

TZM-BL cells were serum starved for 12–24 h then plated overnight in complete media in 96 well plate at 2×10^4^ cells per well. Cells were treated for 1 h with indicated concentrations of inhibitors prior to addition of media alone or 150 ng p24 of HIV_YU2_ HIV_HXB2_ or VSVG or A-MLV-pseudotyped HIV in the presence of 20 ug/ml DEAE-dextran for 3 h at 37°C. After 3 h cells were washed 3 times with PBS and inhibitors were added in fresh media. Following a 24 h incubation cells were lysed and luciferase (luc) units determined. Infected wells and uninfected wells with inhibitor were compared to wells with no inhibitor. For the TZM-Bl assay with lipid analogs serum starved TZM-BL cells were treated with inhibitors for 1 h, then 150 ng of indicated virus was added for 1 h prior to treatment with CPZ or TFP for 1 min or OLA for 5 min. Cells were washed three times with PBS and virus and inhibitors were added back. After 2 h cells were washed two times with PBS and incubated in inhibitor overnight and luc activities were measured.

### Long-Term PBMC Infection and Viability Assay

PBMCs that were isolated and stimulated as previously described [5]. They were plated at 5×10^5^ cells per well in 96 well plate and were treated with 10 µM IMB, 250 nM NIL, or 75 nM DAS for 1 h prior to addition of 150 ng p24 of HIV_HXB2_ in the presence of 20 ug/ml DEAE-dextran for 3 h at 37°C. After 3 h cells were washed three times with PBS and incubated in inhibitor for 24 h. Inhibitors were added back at the same concentration every 24 h for three weeks. 100 ul of supernatant was collected every fourth day and all samples were assayed for p24 antigen content by enzyme-linked immunosorbent assay. Two separate plates were set up under the exact same conditions and one plate was used for p24 measurement and the other was incubated with 20 ul cell viability substrate per 100 ul of sample (Promega, Madison, WI).

### Fusion and Hemifusion Assays

Envelope-mediated and virus-dependent fusion assays were described. Average fusion compared to untreated control reactions were detected by β-galactosidase activity ± standard deviation [5]. To account for any effect of inhibitors on vaccinia virus infection and/or on T7 polymerase function, vCB21R and vPT7-3 co-infected cells were similarly treated with inhibitors. Concentration curves were performed with all of the inhibitors to determine the concentration that resulted in the maximum decrease in fusion without altering vaccinia virus infection or T7 polymerase activity. Hemifusion assays were performed with 2×10^6^ CHO-K1 cells nucleofected with a GFP expression plasmid, and after 24 h infected with vaccinia virus expressing HIV_ADA_ Env or no Env. After 16 h, 4×10^5^ U87.CD4.CCR5.HA cells were added for 3 h, fixed with paraformaldehyde, stained with TRITC-conjugated CTX-555 (EMD), and analyzed on a 510 Meta LSM confocal microscope.

### Statistical Analysis

Fusion and infectivity results were compared using a two-tailed *t*-test. All p values, unless indicated, were <0.03.

## Supporting Information

Figure S1 Abl is activated by HIV-1 Env and pretreatment with inhibitors and siRNA results in specific effects on target molecule. (A) U87.CD4.CCR5 cells were transfected with 200 nM control siRNA or siRNA directed against Trio, Tiam-1, Abl, IRSp53, Wave2, Arp3 and Rac and 48 h later each population of transfected cells was lysed and analyzed by western blot with antibodies to the designated protein or actin. The relative reduction index (RI) is the quotient of the densitometry signal for the target band and that for actin, normalized by the ratio obtained with control siRNA. Data are from 1 of 3 experiments with similar results. The bottom blot depicting Rac levels in cells transfected with the siRNA targeted to control (lane 1), Trio (lane 2), Tiam-1 (lane 3), Abl (lane 4), Rac (lane 5), IRSp53 (lane 6), Wave2 (lane 7), or Arp2/3 (lane 8) demonstrates that the siRNAs have no effect on Rac expression. (B) Abl kinase activity was measured using PAthScan Bcr/Abl activity assay from Cell Signaling. Depicted is western blot analysis of a downstream target of activated Abl kinase, phosphorylated CrkL, and loading control eIF4E from lysates of U87.CD4.CCR5 cells mixed 1∶1 with BSC40 cells expressing no Env (lane 1) or Env from HIV-1 strain ADA (lanes 2–5) at 37°C for 20 min. Cells were pretreated with DMSO alone (NI), 1 uM TAK-779, 10 uM IMB, 500 nM NIL, or 300 nM DAS for 1 hr and during the 20 min incubation with Env-expressing cells. Cell lysates were resolved by 10% SDS-PAGE, transferred to a nitrocellulose membrane, and probed with phosphospecific primary antibody cocktail and anti-rabbit or anti-biotin secondary. Blots shown are from 1 of 3 independent experiments with similar results.
(2.44 MB TIF)

Figure S2 Abl kinase inhibitors decrease Env-induced fusion, virus-dependent fusion and infection of TZM-BL cells with R5 and X4 virus in a concentration dependent manner. Average fusion compared to untreated control reactions was detected by β-gal activity ± standard deviation. (A) Serum starved U87.CD4.CCR5 cells were infected with vCB21R alone, or with vRacV12 overnight, and then treated with DMSO alone, 1 uM TAK-779 or 1, 5, or 10 uM IMB, for 1 h and the inhibitors were also present during the 3 h incubation with HIVUNC (subtracted as background) HIV_ADA_, HIV_YU2_, HIV_89.6_ or HIV_HXB2_ Env-expressing cells. (B) U87.CD4.CCR5 cells were infected overnight with vCB21R or vPT7-3, then mixed (1∶1) in triplicate wells, treated for 1 h with DMSO, 1 uM TAK-779, or 1, 5, or 10 uM IMB, and 100 ng of HIVYU2 added for 3 h at 37°C. Cell fusion was normalized using DMSO treated cells mixed with HIV_YU2_. (C) TZM-BL cells were incubated for 1 h with DMSO, 1 uM TAK-779, 1, 5 or 10 uM IMB, and 150 ng of HIV_YU2_ or 150 ng HIV_HXB2_ per well was added for 3 h, washed, and cells were incubated with inhibitors at 37°C overnight. (D) Serum starved U87.CD4.CCR5 cells were infected with vCB21R alone, or with vRacV12 overnight, then treated with DMSO alone, 125, 250 or 500 nM Nilotinib, or (E) DMSO alone, 75, 150 or 300 nM Dasatinib, for 1 h and the inhibitors were also present during the 3 h incubation with HIV_UNC_ (subtracted as background) HIV_ADA_, HIV_YU2_, HIV_89.6_ or HIV_HXB2_ Env-expressing cells. Data are representative of results from three similar experiments.
(2.88 MB TIF)

Figure S3 Abl kinase is required for X4 Env-dependent and X4 virus-dependent cell-cell fusion. For Env-dependent fusion assays, U87.CD4.CXCR4 cells were infected with vCB21R overnight then treated with DMSO alone, AMD3100, IMB, NIL, or DAS for 1 h and the inhibitors were also present during 3 h incubation with HIV-1 Env-expressing cells and β-gal activity as measured. For X4 virus-dependent fusion assays, U87.CD4.CXCR4 cells were infected overnight with vCB21R or vPT7-3, then mixed (1∶1) in triplicate wells, treated for 1 h with DMSO, AMD3100, IMB, NIL, DAS, and with 250 ng of HIVHXB2 for 5 h at 37°C. β-gal activity was measured and cell fusion was normalized using DMSO treated cells mixed with HIV_HXB2_ as 100%.
(0.89 MB TIF)

Figure S4 Abl kinase inhibitors do not affect surface expression and localization of CD4 and CCR5.GFP. (A) Confocal micrographs of U87.CD4.CCR5 cells treated with DMSO alone, TAK-779 (1 uM), Imatinib (10 uM), Nilotinib (500 nM), Dasatinb (300 nM) and RacGEF Inhibitor (100 uM) for 3 h, and CD (1 uM) for 15 min, fixed and stained with anti-CD4-PE antibodies (Sigma, red) and counterstained with TO-PRO3 (blue). The green GFP signal and red PE signal have been merged to show areas of colocalization (yellow). Images are from 1 of 3 experiments with similar results. Images were collected using an oil objective (magnification X63). The Nilotinib panel is a consolidation of 2 separate images from the same experiment. (B) U87.CD4.CCR5 cells were incubated with no inhibitor, DMSO alone, TAK-779 (1 uM), Imatinib (10 uM), Nilotinib (500 nM), Dasatinb (300 nM) and RacGEF Inhibitor (100 uM) for 3 h and detached by treatment with 5 mM EDTA. U87 cells, and untreated and treated U87.CD4.CCR5.GFP cells, were stained with anti-CCR5 (R&D) or anti-CD4 antibodies (Sigma), and goat anti-mouse PE conjugated antibody. Cells were analyzed on a FACS Calibur flow cytometer. Unlabeled U87.CD4.CCR5.GFP cells were used to compensate for GFP. Data are expressed as percentage of surface expression based on DMSO treated cells as 100%.
(8.02 MB TIF)

Figure S5 Treatment of PBLs with Abl kinase inhibitors for 3 weeks blocks infection of X4 HIV_HXB2_ virus without significant cell toxicity. PBLs were treated for 1 h with no inhibitor, DMSO, 10 µM IMB, 250 nM NIL, or 75 nM DAS for 1 h prior to and during infection with 200 ng HIV_HXB2_ virus for 3 h. After 3 h virus and inhibitor were washed off, inhibitor was added back and cells were incubated at 37° for 24 h. Every 24 h for 21 days inhibitor was added back at the same concentration. At day 21, supernatants were harvested and p24 antigen content was measured or cells were harvested and cell viability was assayed. P24 content and cell viability were normalized using no inhibitor treated cells infected with HIV_HXB2_ virus as 100%.
(1.14 MB TIF)

Figure S6 The membrane curving lipid analogs CPZ, TFP, and OLA have no effect on infection with HIV-1ΔEnv, but positive membrane curving lipid analogs overcome Abl kinase induced inhibition of R5 HIV_YU2_ virus-dependent cell-cell fusion and X4 HIV_HXB2_ virus-cell fusion. (A) TZM-BL cells were treated with DMSO, TAK-779, IMB, NIL, or DAS for 1 h prior to 1 h incubation with 150 ng of HIV-1ΔEnv (B) TZM-BL cells were treated with DMSO, or NIL for 1 hr prior to 1 h incubation with 150 ng HIV_HXB2_ virus (C) U87.CD4.CCR5 cells infected with vCB21R or vPT7-3 overnight, then mixed (1∶1) in triplicate wells of 96 well plate were treated with DMSO, TAK-779, IMB, NIL, or DAS for 1 h prior to 1 h incubation with 100 ng of HIV_YU2_. After 1 h indicated lipid analogs were added for 1–5 min (A) cells were washed, and virus and inhibitors were added back for (A, C) 3 h or (B) 90 min and (A) cells were washed and incubated in inhibitor overnight and luc activities were measured or (B) BlaM activity was measured or (C) β-gal activity was measured. Data are representative of results from three similar experiments. Cell fusion was normalized using DMSO treated cells incubated with HIV_YU2_ as 100%.
(0.67 MB TIF)

Figure S7 Abl-kinase inhibitors block fusion at a post-hemifusion step. CHO-K1 cells that do not express GM1 were transfected with a GFP expressing plasmid, and 24 h later infected with wildtype vaccinia virus or vaccinia virus expressing HIV_ADA_. After another 16 h, CHO-K1 cells were overlayed for 3 h with U87.CD4.CCR5 cells pre-treated for 1 h with DMSO, 1 µM TAK-779, or 10 µM IMB. Cells were fixed and stained with TRITC-conjugated CTX (CTX-555, red), and counterstained with TO-PRO3 (blue). Images were collected using an oil objective (magnification X63). Images were cropped but relative cell size was maintained.
(7.02 MB TIF)

Figure S8 IMB induced arrest at hemifusion step is overcome with membrane curving lipid analog CPZ. U87.CD4.CCR5 cells were pre-treated for 1 h with DMSO, 1 µM TAK-779, or 10 µM IMB, incubated with CHO-K1 cells expressing no Env or HIV_ADA_ Env for 1 h, treated with CPZ for 1 min, then washed and inhibitors were added back for additional 2 h incubation at 37°. Cells were fixed and stained with TRITC-conjugated CTX (CTX-555, red), and counterstained with TO-PRO3 (blue). Images were collected using an oil objective (magnification X63). Images were cropped but relative cell size was maintained.
(7.06 MB TIF)

Figure S9 A-MLV Env- and VSV-G-induced cell-cell fusion is unaffected by IMB or CPZ. U87.CD4.CCR5 cells were pre-treated for 1 h with DMSO or 10 µM IMB, incubated with CHO-K1 cells expressing A-MLV Env or VSV-G for 1 h, treated with CPZ for 1 min, then washed and inhibitors were added back for additional 2 h incubation at 37°. Cells were fixed and stained with TRITC-conjugated CTX (CTX-555, red), and counterstained with TO-PRO3 (blue). Images were collected using an oil objective (magnification X63). Images were cropped but relative cell size was maintained.
(9.94 MB TIF)

Figure S10 The actin monomer sequestering drugs LA and LB and the dynamin inhibitor DYN block HIV-1 Env-dependent cell-cell fusion, virus-dependent cell-cell fusion, virus-cell fusion and infection. (A) Serum starved U87.CD4.CCR5 cells or U87.CD4.CXCR4 cells were treated with DMSO alone, 1 µM TAK-779, 1 µM AMD3100, 1 µM LA, 5 µM LB, or 80 µM DYN for 1 h and these cells were used in an (A) Env-dependent cell-cell fusion assay or a (B) virus-dependent cell-cell fusion assay and β-gal activity was measured. Cell fusion was normalized using DMSO treated cells mixed with HIV_HXB2_ or HIV_YU2_ (A) Env-expressing cells or (B) virus as 100%. (C & D) TZM-BL cells were incubated for 1 h with DMSO, 1 µM TAK-779, 1 µM AMD3100, 1 µM LA, 5 µM LB, 80 µM DYN, then 150 ng of (A) HIV_HXB2_ HIV_ADA_ or HIV_YU2_ virus was added for 90 min and BlaM activity was measured or (B) 150 ng HIV_YU2_ or HIVHXB2 virus was added for 3 h, washed, and cells were incubated with inhibitors at 37°C overnight. Virus cell fusion and infection were normalized using DMSO treated cells infected with (C) HIV_HXB2_ or (D) HIV_YU2_ virus as 100%. Data are representative of results from three similar experiments performed in triplicate.
(1.65 MB TIF)

Figure S11 The dynamin inhibitor DYN blocks A-MLV-Env and VSV-G-mediated infection to a greater extent than HIV-1 Env mediated infection. TZM-BL cells were incubated for 1 h with DMSO, 1 µM TAK-779, 1 µM AMD3100, 40 µM DYN, 80 µM DYN, or 160 µM DYN, then 150 ng of HIV_HXB2_ HIV_YU2_, A-MLV-Env-HIV-1, or VSV-G-HIV-1 was added for 3 h, washed, and cells were incubated with inhibitors at 37°C overnight. Virus cell fusion and infection were normalized using DMSO treated cells infected with HIV-1 virus as 100%. Data are representative of results from three similar experiments performed in triplicate.
(1.29 MB TIF)

Table S1 Quantification of cell-cell hemifusion assay.
(0.03 MB DOC)
