# Supplementary figures and images for: Role of Abl Kinase and the Wave2 Signaling Complex in HIV-1 Entry at a Post-Hemifusion Step
Source: PLoS Pathog. 2010 Jun 17;6(6):e1000956. doi: 10.1371/journal.ppat.1000956 (PMC2887473; doi:10.1371/journal.ppat.1000956)

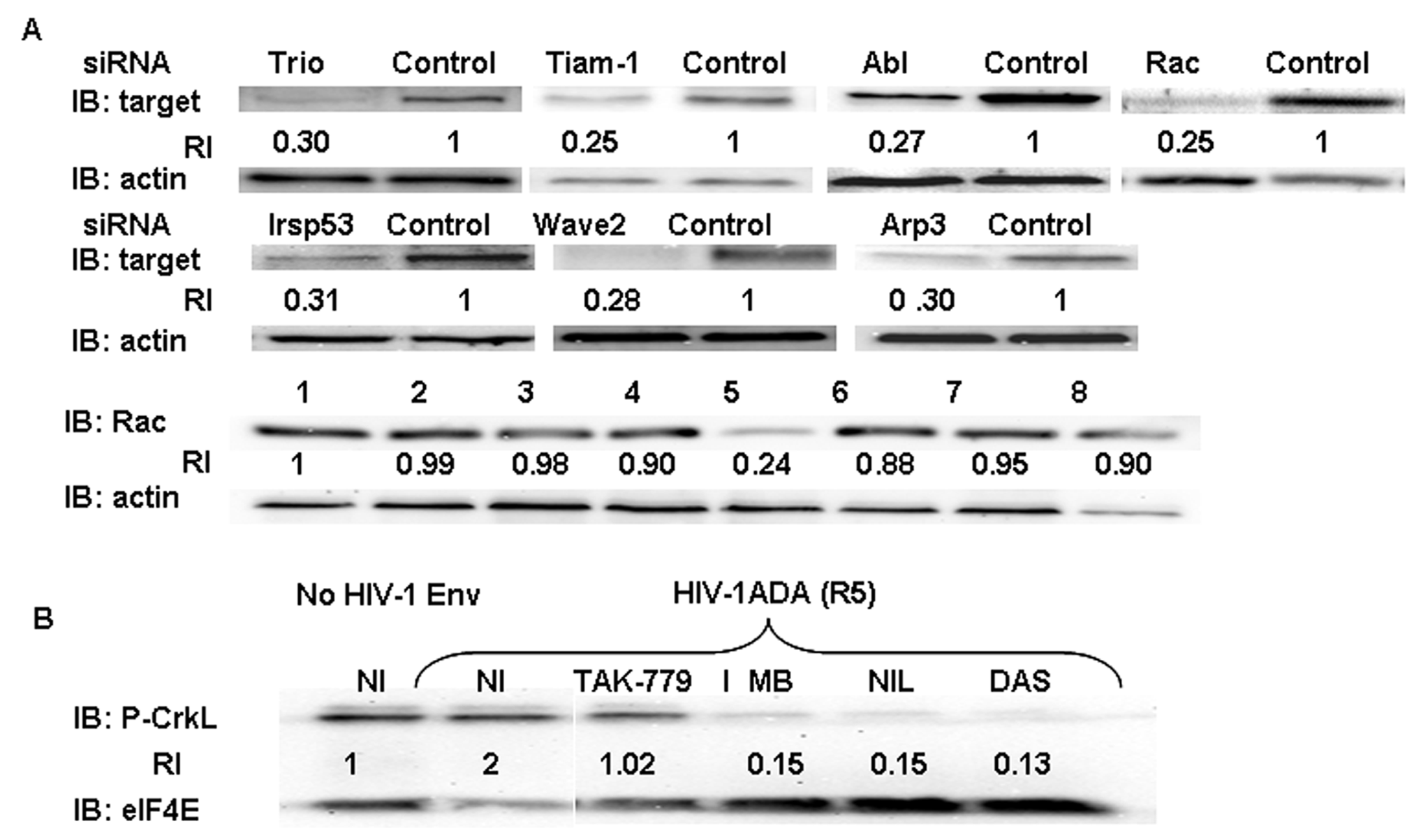

Supplement: Figure S1 — Abl is activated by HIV-1 Env and pretreatment with inhibitors and siRNA results in specific effects on target molecule. (A) U87.CD4.CCR5 cells were transfected with 200 nM control siRNA or siRNA directed against Trio, Tiam-1, Abl, IRSp53, Wave2, Arp3 and Rac and 48 h later each population of transfected cells was lysed and analyzed by western blot with antibodies to the designated protein or actin. The relative reduction index (RI) is the quotient of the densitometry signal for the target band and that for actin, normalized by the ratio obtained with control siRNA. Data are from 1 of 3 experiments with similar results. The bottom blot depicting Rac levels in cells transfected with the siRNA targeted to control (lane 1), Trio (lane 2), Tiam-1 (lane 3), Abl (lane 4), Rac (lane 5), IRSp53 (lane 6), Wave2 (lane 7), or Arp2/3 (lane 8) demonstrates that the siRNAs have no effect on Rac expression. (B) Abl kinase activity was measured using PAthScan Bcr/Abl activity assay from Cell Signaling. Depicted is western blot analysis of a downstream target of activated Abl kinase, phosphorylated CrkL, and loading control eIF4E from lysates of U87.CD4.CCR5 cells mixed 1∶1 with BSC40 cells expressing no Env (lane 1) or Env from HIV-1 strain ADA (lanes 2–5) at 37°C for 20 min. Cells were pretreated with DMSO alone (NI), 1 uM TAK-779, 10 uM IMB, 500 nM NIL, or 300 nM DAS for 1 hr and during the 20 min incubation with Env-expressing cells. Cell lysates were resolved by 10% SDS-PAGE, transferred to a nitrocellulose membrane, and probed with phosphospecific primary antibody cocktail and anti-rabbit or anti-biotin secondary. Blots shown are from 1 of 3 independent experiments with similar results. (2.44 MB TIF) [file ppat.1000956.s001.tif]

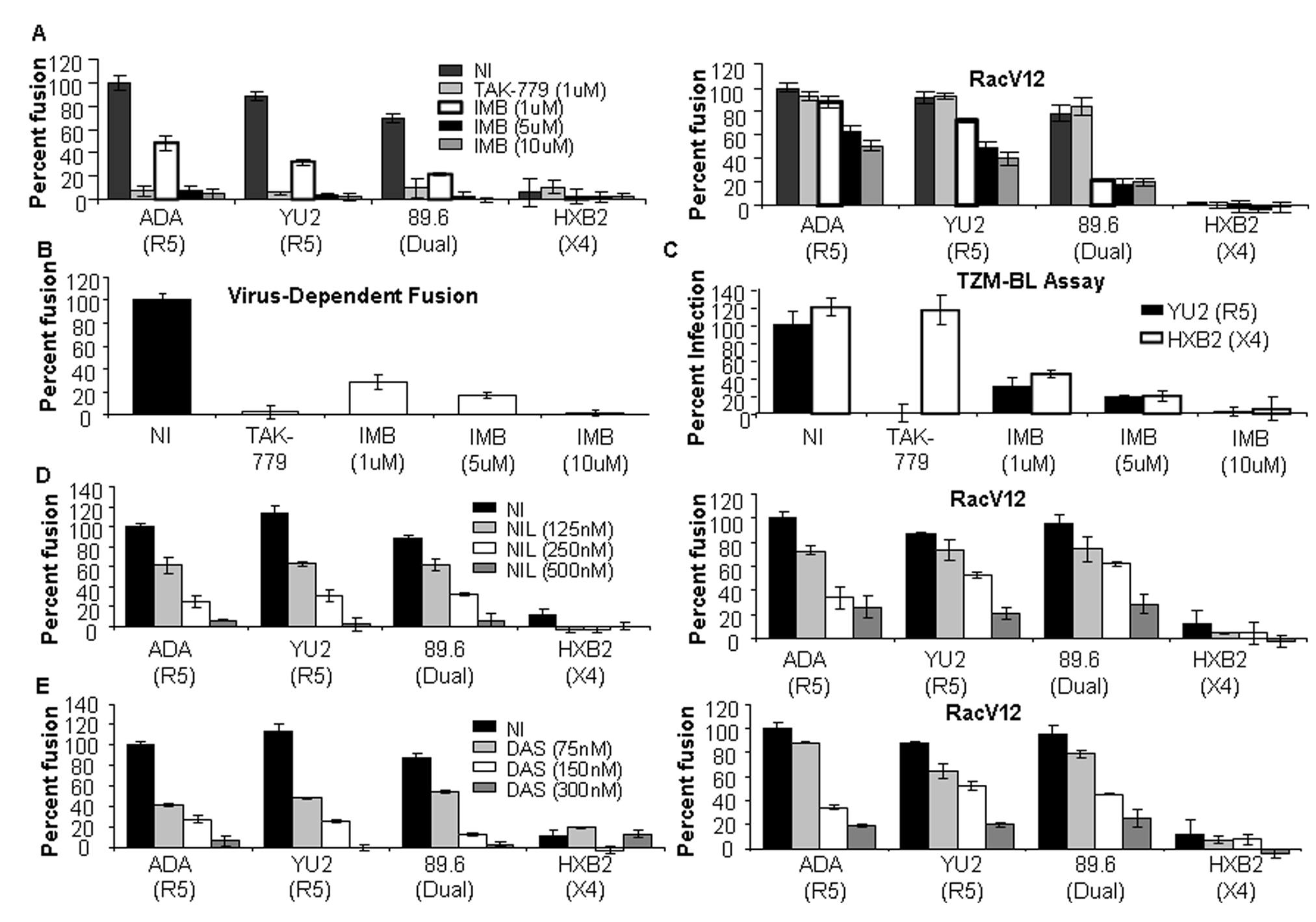

Supplement: Figure S2 — Abl kinase inhibitors decrease Env-induced fusion, virus-dependent fusion and infection of TZM-BL cells with R5 and X4 virus in a concentration dependent manner. Average fusion compared to untreated control reactions was detected by β-gal activity ± standard deviation. (A) Serum starved U87.CD4.CCR5 cells were infected with vCB21R alone, or with vRacV12 overnight, and then treated with DMSO alone, 1 uM TAK-779 or 1, 5, or 10 uM IMB, for 1 h and the inhibitors were also present during the 3 h incubation with HIVUNC (subtracted as background) HIVADA, HIVYU2, HIV89.6 or HIVHXB2 Env-expressing cells. (B) U87.CD4.CCR5 cells were infected overnight with vCB21R or vPT7-3, then mixed (1∶1) in triplicate wells, treated for 1 h with DMSO, 1 uM TAK-779, or 1, 5, or 10 uM IMB, and 100 ng of HIVYU2 added for 3 h at 37°C. Cell fusion was normalized using DMSO treated cells mixed with HIVYU2. (C) TZM-BL cells were incubated for 1 h with DMSO, 1 uM TAK-779, 1, 5 or 10 uM IMB, and 150 ng of HIVYU2 or 150 ng HIVHXB2 per well was added for 3 h, washed, and cells were incubated with inhibitors at 37°C overnight. (D) Serum starved U87.CD4.CCR5 cells were infected with vCB21R alone, or with vRacV12 overnight, then treated with DMSO alone, 125, 250 or 500 nM Nilotinib, or (E) DMSO alone, 75, 150 or 300 nM Dasatinib, for 1 h and the inhibitors were also present during the 3 h incubation with HIVUNC (subtracted as background) HIVADA, HIVYU2, HIV89.6 or HIVHXB2 Env-expressing cells. Data are representative of results from three similar experiments. (2.88 MB TIF) [file ppat.1000956.s002.tif]

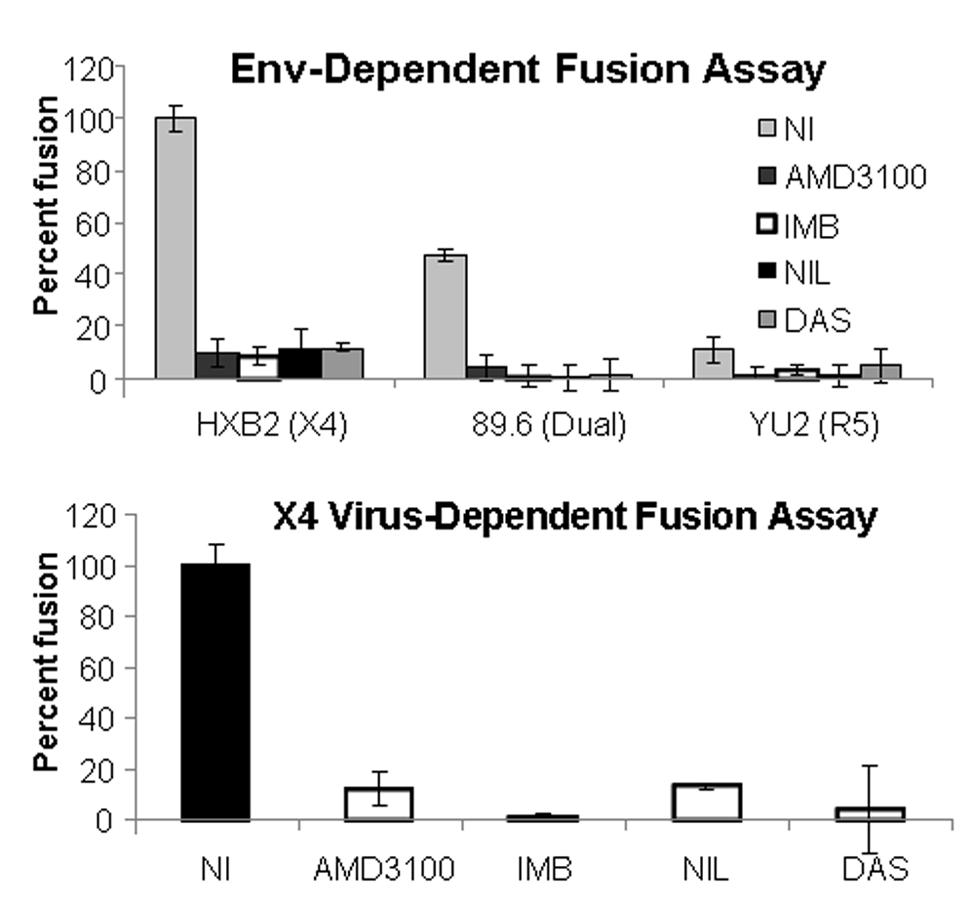

Supplement: Figure S3 — Abl kinase is required for X4 Env-dependent and X4 virus-dependent cell-cell fusion. For Env-dependent fusion assays, U87.CD4.CXCR4 cells were infected with vCB21R overnight then treated with DMSO alone, AMD3100, IMB, NIL, or DAS for 1 h and the inhibitors were also present during 3 h incubation with HIV-1 Env-expressing cells and β-gal activity as measured. For X4 virus-dependent fusion assays, U87.CD4.CXCR4 cells were infected overnight with vCB21R or vPT7-3, then mixed (1∶1) in triplicate wells, treated for 1 h with DMSO, AMD3100, IMB, NIL, DAS, and with 250 ng of HIVHXB2 for 5 h at 37°C. β-gal activity was measured and cell fusion was normalized using DMSO treated cells mixed with HIVHXB2 as 100%. (0.89 MB TIF) [file ppat.1000956.s003.tif]

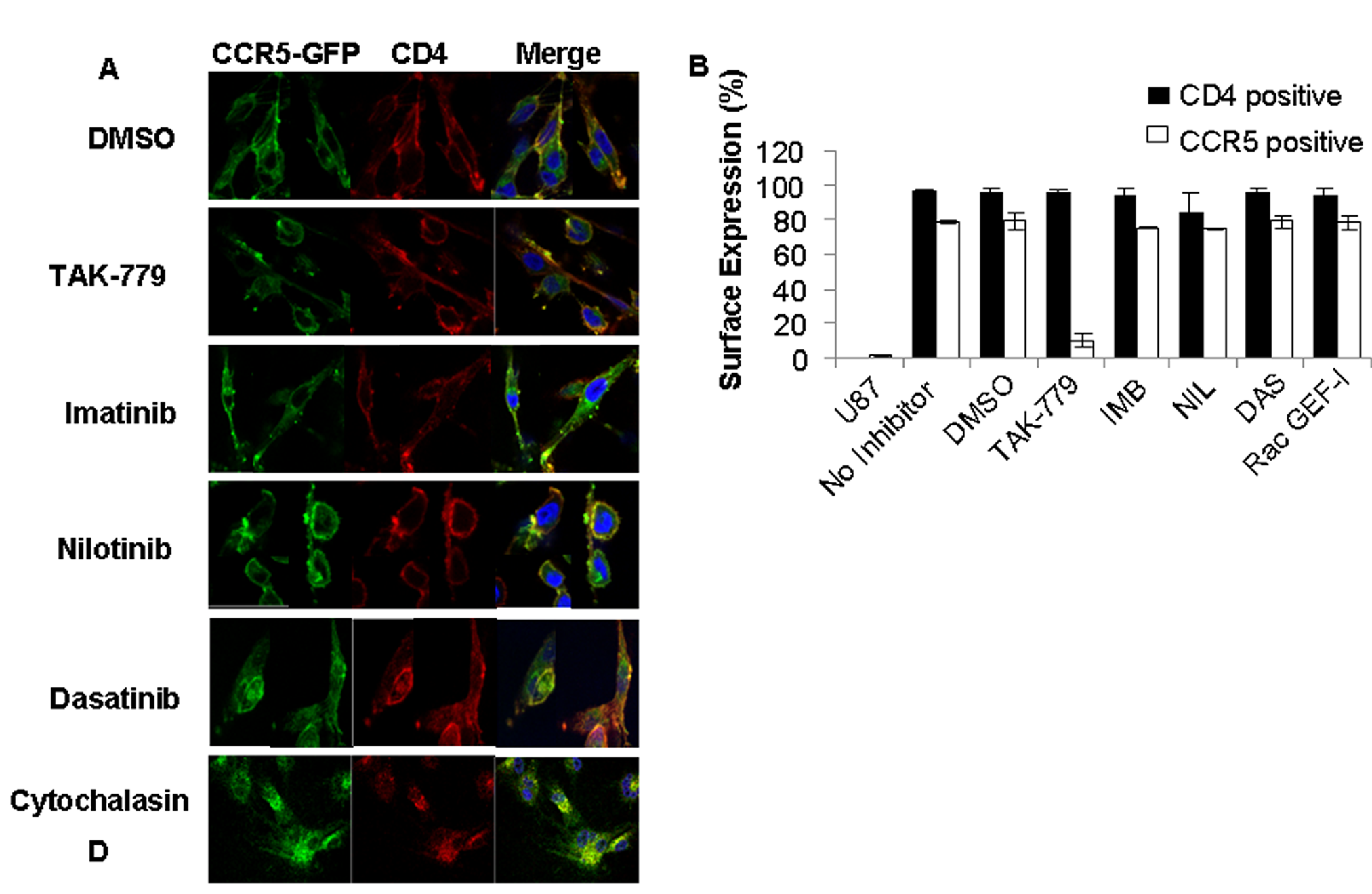

Supplement: Figure S4 — Abl kinase inhibitors do not affect surface expression and localization of CD4 and CCR5.GFP. (A) Confocal micrographs of U87.CD4.CCR5 cells treated with DMSO alone, TAK-779 (1 uM), Imatinib (10 uM), Nilotinib (500 nM), Dasatinb (300 nM) and RacGEF Inhibitor (100 uM) for 3 h, and CD (1 uM) for 15 min, fixed and stained with anti-CD4-PE antibodies (Sigma, red) and counterstained with TO-PRO3 (blue). The green GFP signal and red PE signal have been merged to show areas of colocalization (yellow). Images are from 1 of 3 experiments with similar results. Images were collected using an oil objective (magnification X63). The Nilotinib panel is a consolidation of 2 separate images from the same experiment. (B) U87.CD4.CCR5 cells were incubated with no inhibitor, DMSO alone, TAK-779 (1 uM), Imatinib (10 uM), Nilotinib (500 nM), Dasatinb (300 nM) and RacGEF Inhibitor (100 uM) for 3 h and detached by treatment with 5 mM EDTA. U87 cells, and untreated and treated U87.CD4.CCR5.GFP cells, were stained with anti-CCR5 (R&D) or anti-CD4 antibodies (Sigma), and goat anti-mouse PE conjugated antibody. Cells were analyzed on a FACS Calibur flow cytometer. Unlabeled U87.CD4.CCR5.GFP cells were used to compensate for GFP. Data are expressed as percentage of surface expression based on DMSO treated cells as 100%. (8.02 MB TIF) [file ppat.1000956.s004.tif]

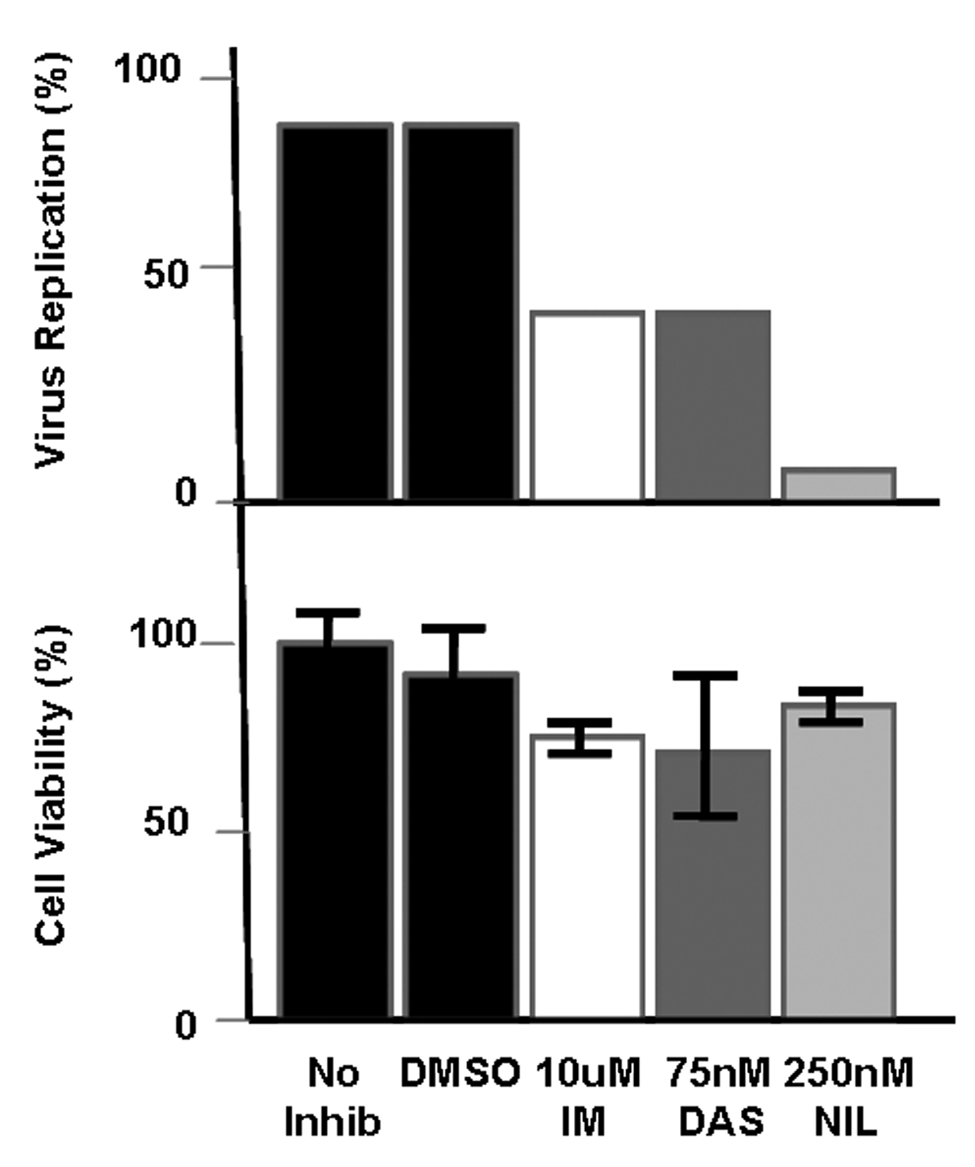

Supplement: Figure S5 — Treatment of PBLs with Abl kinase inhibitors for 3 weeks blocks infection of X4 HIVHXB2 virus without significant cell toxicity. PBLs were treated for 1 h with no inhibitor, DMSO, 10 µM IMB, 250 nM NIL, or 75 nM DAS for 1 h prior to and during infection with 200 ng HIVHXB2 virus for 3 h. After 3 h virus and inhibitor were washed off, inhibitor was added back and cells were incubated at 37° for 24 h. Every 24 h for 21 days inhibitor was added back at the same concentration. At day 21, supernatants were harvested and p24 antigen content was measured or cells were harvested and cell viability was assayed. P24 content and cell viability were normalized using no inhibitor treated cells infected with HIVHXB2 virus as 100%. (1.14 MB TIF) [file ppat.1000956.s005.tif]

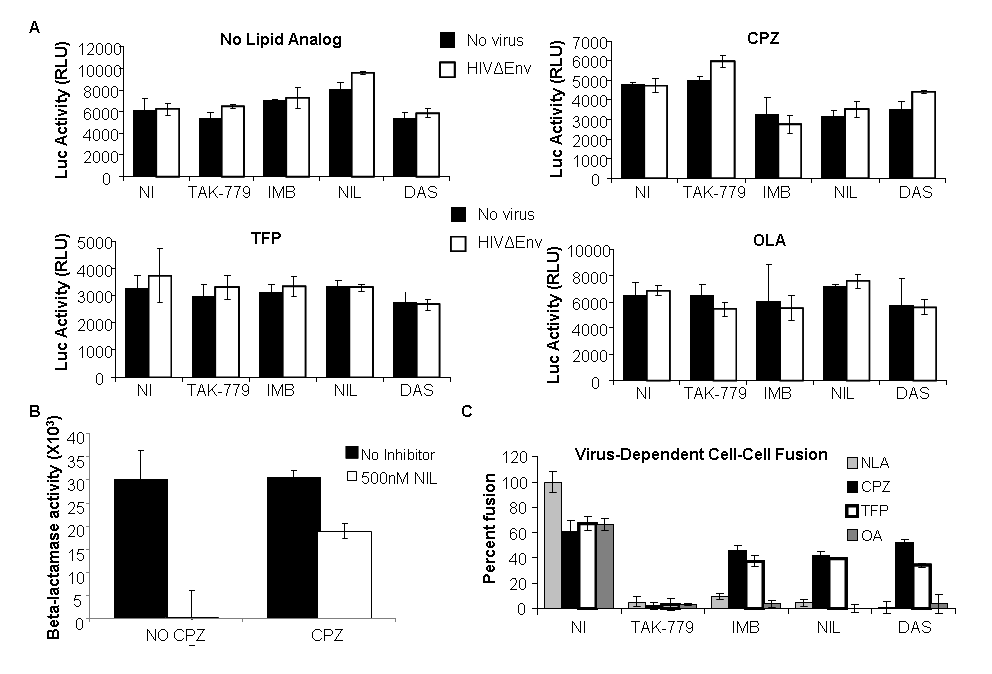

Supplement: Figure S6 — The membrane curving lipid analogs CPZ, TFP, and OLA have no effect on infection with HIV-1ΔEnv, but positive membrane curving lipid analogs overcome Abl kinase induced inhibition of R5 HIVYU2 virus-dependent cell-cell fusion and X4 HIVHXB2 virus-cell fusion. (A) TZM-BL cells were treated with DMSO, TAK-779, IMB, NIL, or DAS for 1 h prior to 1 h incubation with 150 ng of HIV-1ΔEnv (B) TZM-BL cells were treated with DMSO, or NIL for 1 hr prior to 1 h incubation with 150 ng HIVHXB2 virus (C) U87.CD4.CCR5 cells infected with vCB21R or vPT7-3 overnight, then mixed (1∶1) in triplicate wells of 96 well plate were treated with DMSO, TAK-779, IMB, NIL, or DAS for 1 h prior to 1 h incubation with 100 ng of HIVYU2. After 1 h indicated lipid analogs were added for 1–5 min (A) cells were washed, and virus and inhibitors were added back for (A, C) 3 h or (B) 90 min and (A) cells were washed and incubated in inhibitor overnight and luc activities were measured or (B) BlaM activity was measured or (C) β-gal activity was measured. Data are representative of results from three similar experiments. Cell fusion was normalized using DMSO treated cells incubated with HIVYU2 as 100%. (0.67 MB TIF) [file ppat.1000956.s006.tif]

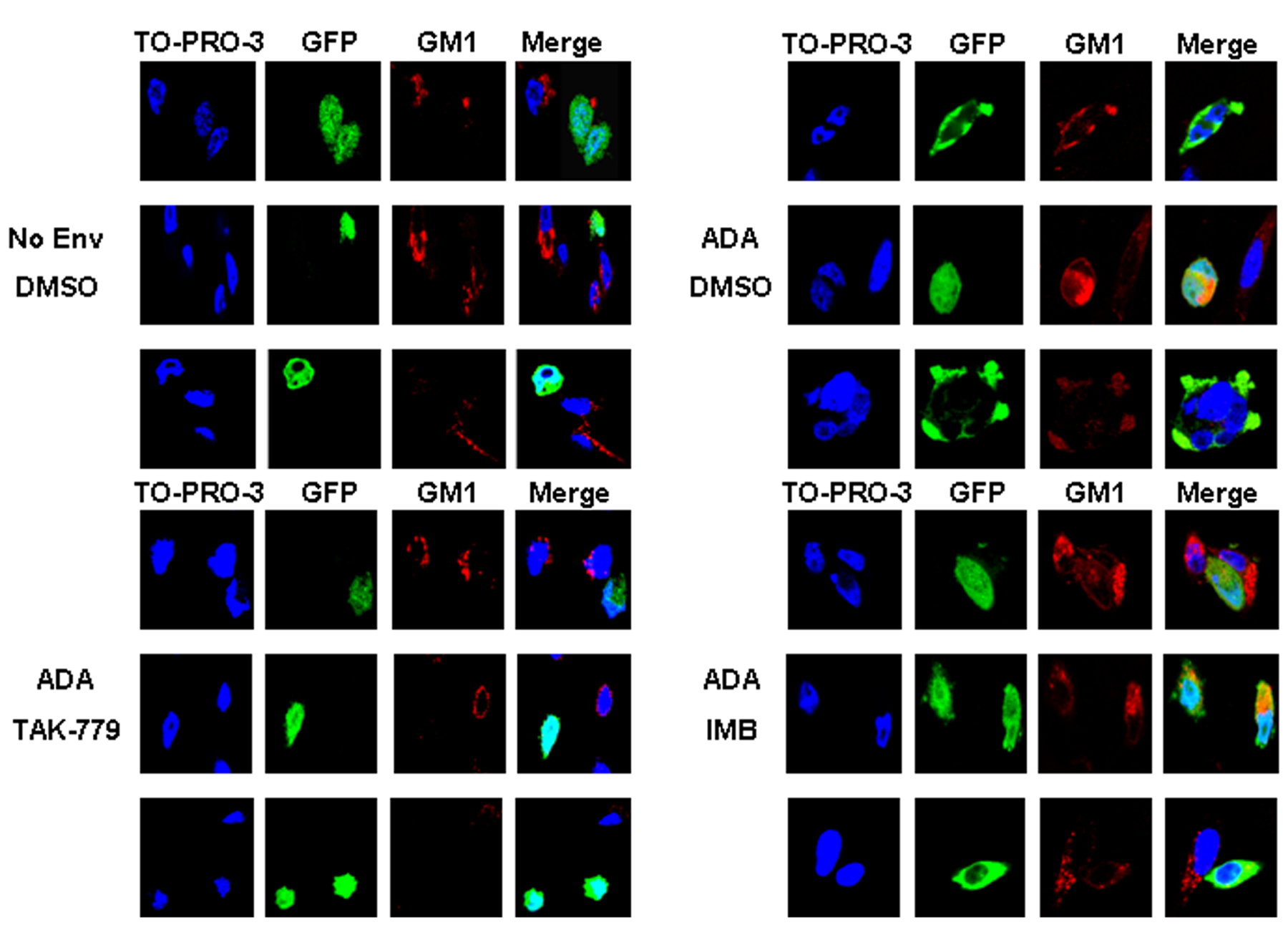

Supplement: Figure S7 — Abl-kinase inhibitors block fusion at a post-hemifusion step. CHO-K1 cells that do not express GM1 were transfected with a GFP expressing plasmid, and 24 h later infected with wildtype vaccinia virus or vaccinia virus expressing HIVADA. After another 16 h, CHO-K1 cells were overlayed for 3 h with U87.CD4.CCR5 cells pre-treated for 1 h with DMSO, 1 µM TAK-779, or 10 µM IMB. Cells were fixed and stained with TRITC-conjugated CTX (CTX-555, red), and counterstained with TO-PRO3 (blue). Images were collected using an oil objective (magnification X63). Images were cropped but relative cell size was maintained. (7.02 MB TIF) [file ppat.1000956.s007.tif]

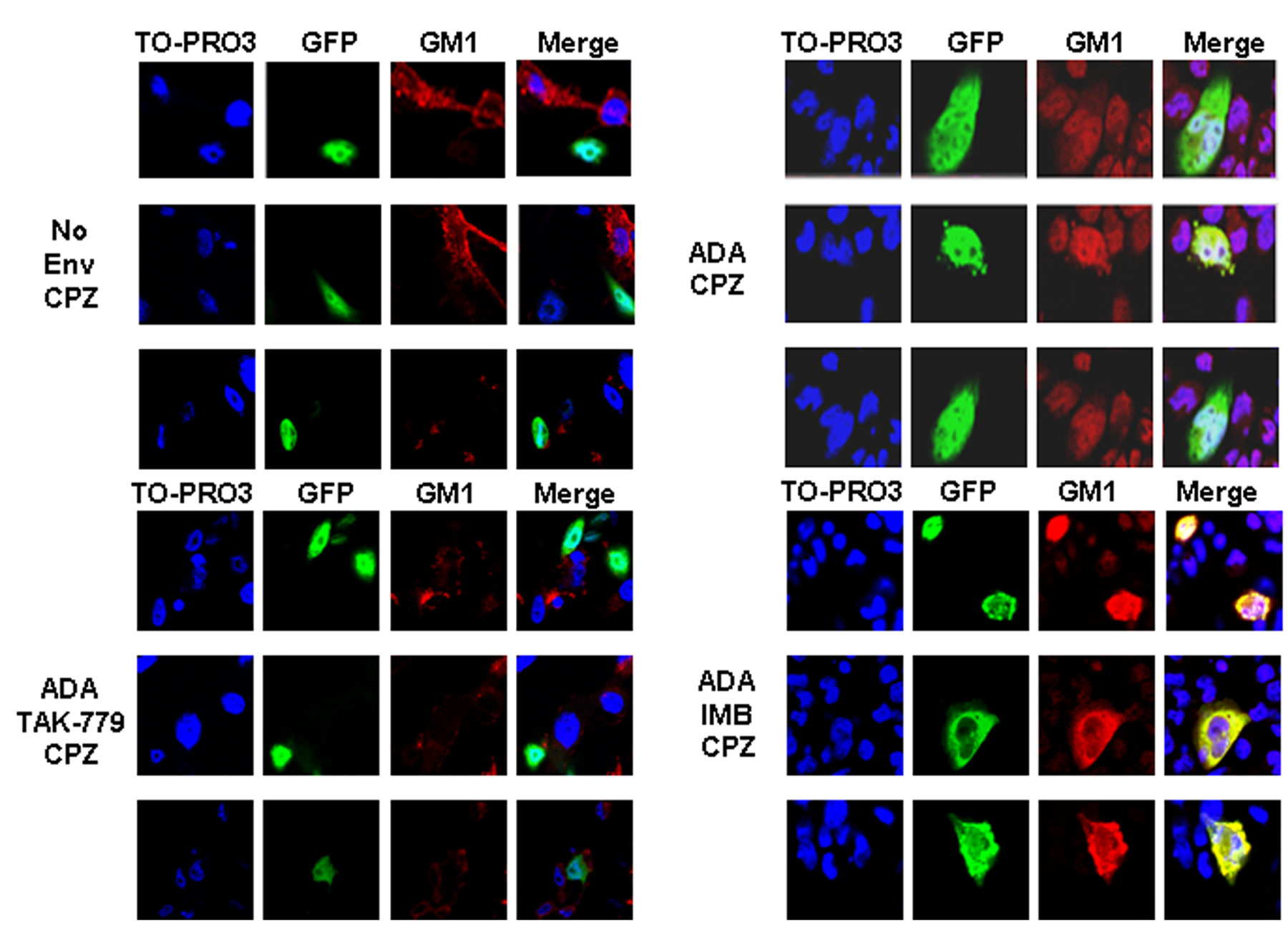

Supplement: Figure S8 — IMB induced arrest at hemifusion step is overcome with membrane curving lipid analog CPZ. U87.CD4.CCR5 cells were pre-treated for 1 h with DMSO, 1 µM TAK-779, or 10 µM IMB, incubated with CHO-K1 cells expressing no Env or HIVADA Env for 1 h, treated with CPZ for 1 min, then washed and inhibitors were added back for additional 2 h incubation at 37°. Cells were fixed and stained with TRITC-conjugated CTX (CTX-555, red), and counterstained with TO-PRO3 (blue). Images were collected using an oil objective (magnification X63). Images were cropped but relative cell size was maintained. (7.06 MB TIF) [file ppat.1000956.s008.tif]

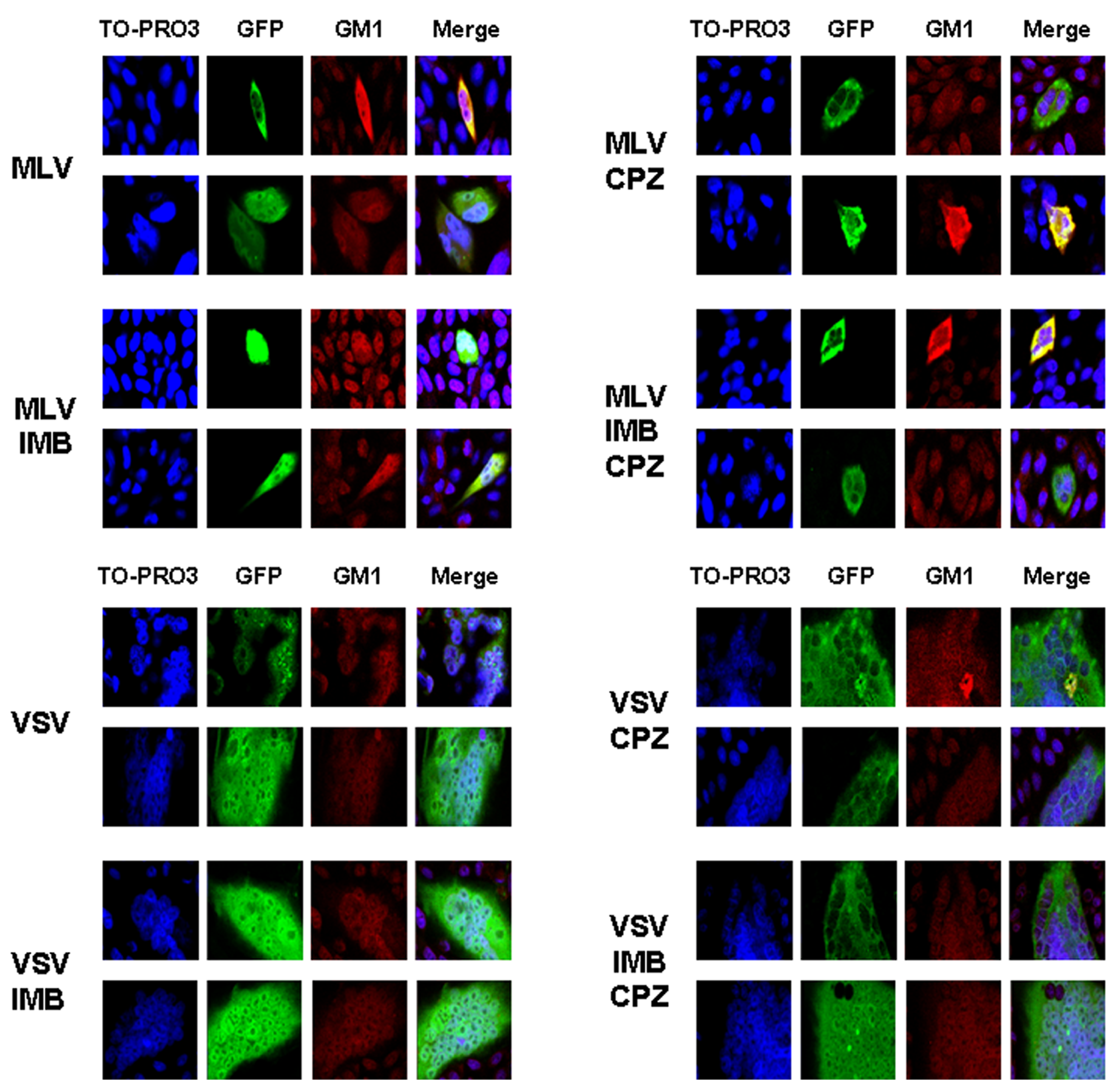

Supplement: Figure S9 — A-MLV Env- and VSV-G-induced cell-cell fusion is unaffected by IMB or CPZ. U87.CD4.CCR5 cells were pre-treated for 1 h with DMSO or 10 µM IMB, incubated with CHO-K1 cells expressing A-MLV Env or VSV-G for 1 h, treated with CPZ for 1 min, then washed and inhibitors were added back for additional 2 h incubation at 37°. Cells were fixed and stained with TRITC-conjugated CTX (CTX-555, red), and counterstained with TO-PRO3 (blue). Images were collected using an oil objective (magnification X63). Images were cropped but relative cell size was maintained. (9.94 MB TIF) [file ppat.1000956.s009.tif]

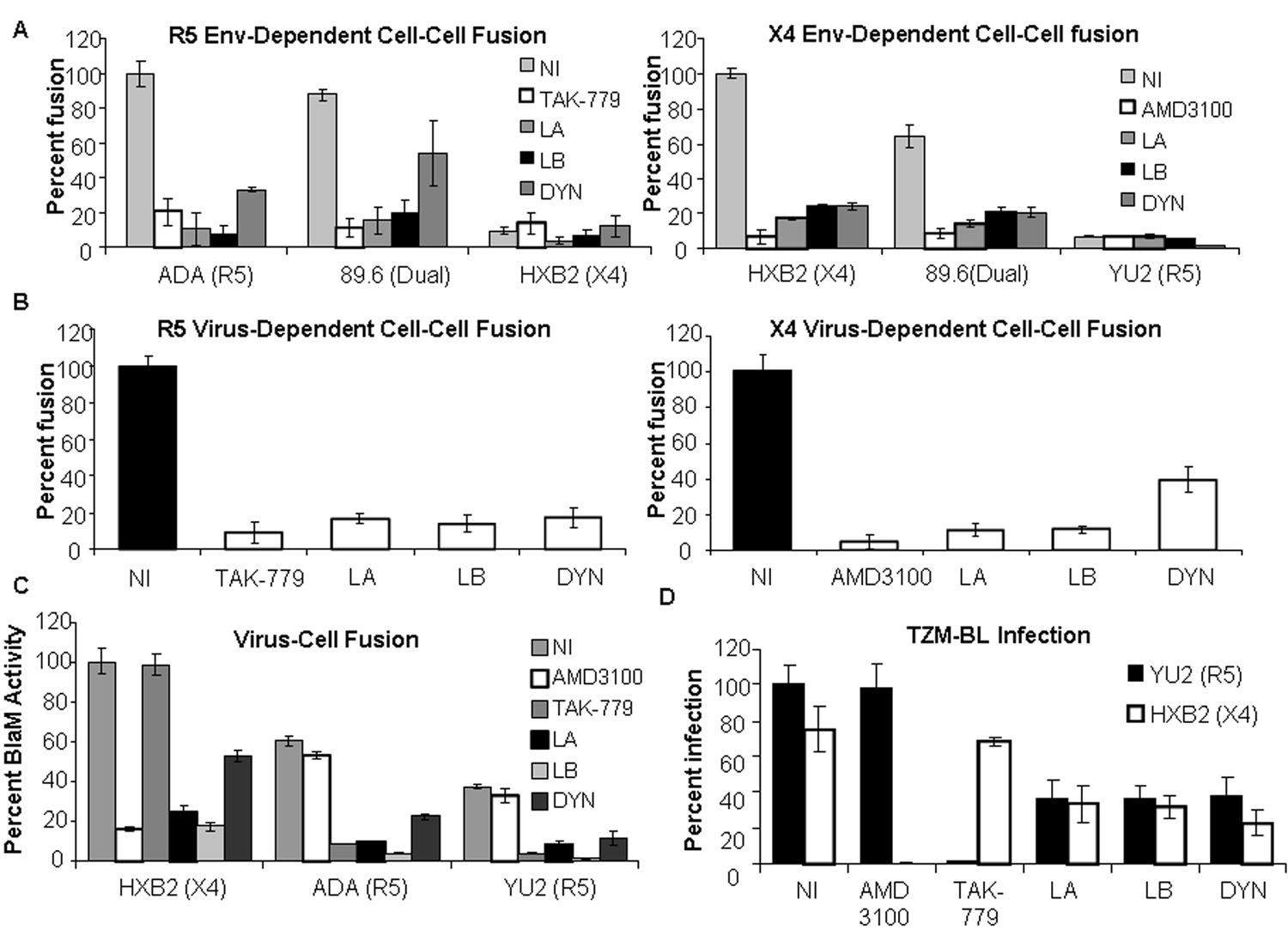

Supplement: Figure S10 — The actin monomer sequestering drugs LA and LB and the dynamin inhibitor DYN block HIV-1 Env-dependent cell-cell fusion, virus-dependent cell-cell fusion, virus-cell fusion and infection. (A) Serum starved U87.CD4.CCR5 cells or U87.CD4.CXCR4 cells were treated with DMSO alone, 1 µM TAK-779, 1 µM AMD3100, 1 µM LA, 5 µM LB, or 80 µM DYN for 1 h and these cells were used in an (A) Env-dependent cell-cell fusion assay or a (B) virus-dependent cell-cell fusion assay and β-gal activity was measured. Cell fusion was normalized using DMSO treated cells mixed with HIVHXB2 or HIVYU2 (A) Env-expressing cells or (B) virus as 100%. (C & D) TZM-BL cells were incubated for 1 h with DMSO, 1 µM TAK-779, 1 µM AMD3100, 1 µM LA, 5 µM LB, 80 µM DYN, then 150 ng of (A) HIVHXB2 HIVADA or HIVYU2 virus was added for 90 min and BlaM activity was measured or (B) 150 ng HIVYU2 or HIVHXB2 virus was added for 3 h, washed, and cells were incubated with inhibitors at 37°C overnight. Virus cell fusion and infection were normalized using DMSO treated cells infected with (C) HIVHXB2 or (D) HIVYU2 virus as 100%. Data are representative of results from three similar experiments performed in triplicate. (1.65 MB TIF) [file ppat.1000956.s010.tif]

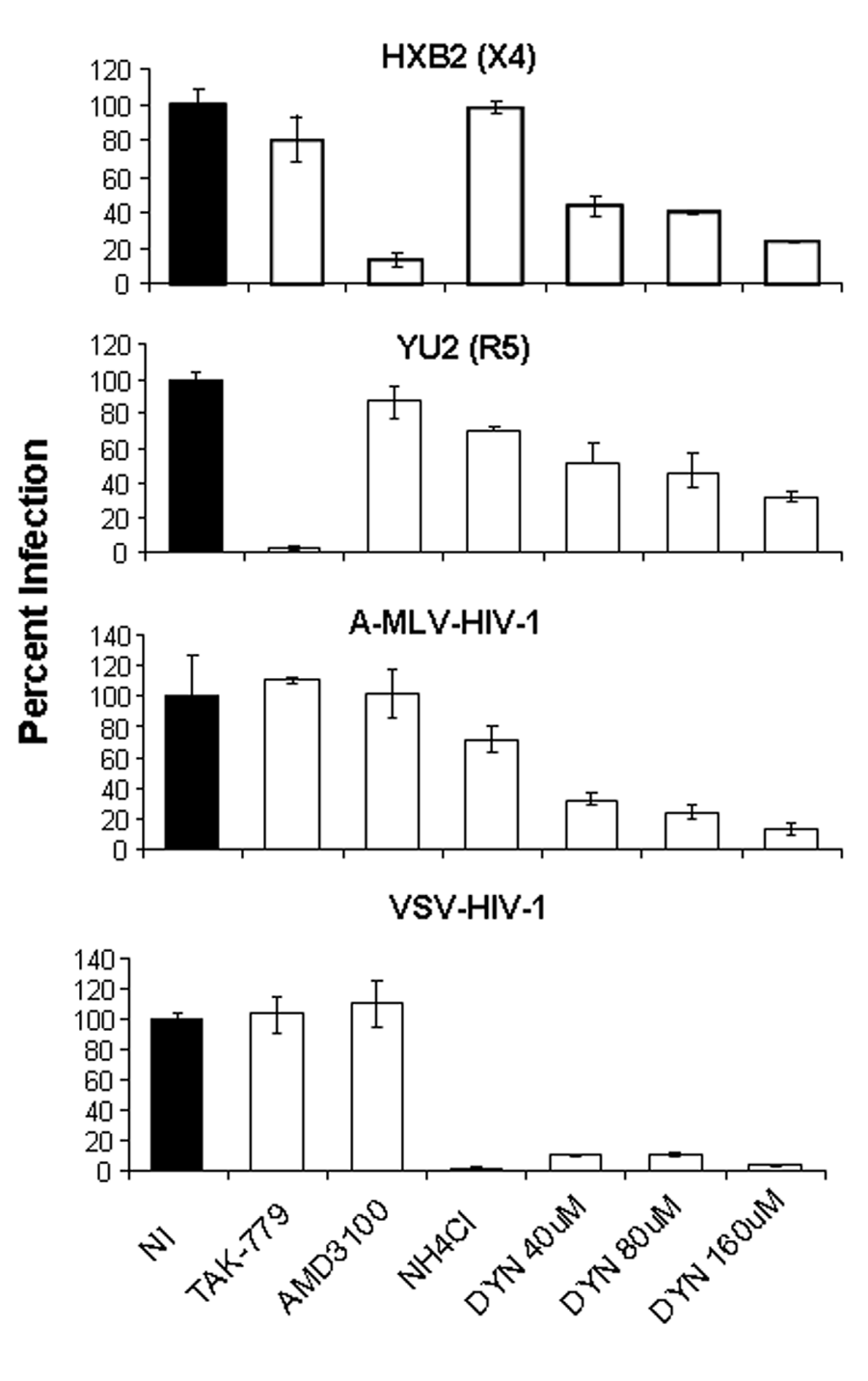

Supplement: Figure S11 — The dynamin inhibitor DYN blocks A-MLV-Env and VSV-G-mediated infection to a greater extent than HIV-1 Env mediated infection. TZM-BL cells were incubated for 1 h with DMSO, 1 µM TAK-779, 1 µM AMD3100, 40 µM DYN, 80 µM DYN, or 160 µM DYN, then 150 ng of HIVHXB2 HIVYU2, A-MLV-Env-HIV-1, or VSV-G-HIV-1 was added for 3 h, washed, and cells were incubated with inhibitors at 37°C overnight. Virus cell fusion and infection were normalized using DMSO treated cells infected with HIV-1 virus as 100%. Data are representative of results from three similar experiments performed in triplicate. (1.29 MB TIF) [file ppat.1000956.s011.tif]
