# Supplementary material for: Role of Abl Kinase and the Wave2 Signaling Complex in HIV-1 Entry at a Post-Hemifusion Step
Source: PLoS Pathog. 2010 Jun 17;6(6):e1000956. doi: 10.1371/journal.ppat.1000956 (PMC2887473; doi:10.1371/journal.ppat.1000956)
Supplement: Table S1 — Quantification of cell-cell hemifusion assay. (0.03 MB DOC) [file ppat.1000956.s012.doc]

| **HIV Env** | **Inhibitor** | **% hemifusion ± SD** | **% fusion ± SD** |
| --- | --- | --- | --- |
| None (n=68) | DMSO | 0 | 0 |
| ADA (R5) (n=68) | DMSO | 22.3 ± 4.9 | 75.5 ± 6.2 |
| ADA (R5) (n=68) | TAK-779 | 7 ± 4.8 | 0 |
| ADA (R5) (n=68) | Imatinib | 83.1 ±10.9 | 14.3 ± 5.1 |

Table S1. Quantification of cell-cell hemifusion assay
